# The TetR-like regulator Sco4385 and Crp-like regulator Sco3571 modulate heterologous production of antibiotics in *Streptomyces coelicolor* M512

**DOI:** 10.1128/aem.02315-24

**Published:** 2025-04-04

**Authors:** Sarah Wilcken, Panagiota-Hanna Koutsandrea, Tomke Bakker, Andreas Kulik, Tim Orthwein, Mirita Franz-Wachtel, Theresa Harbig, Kay Katja Nieselt, Karl Forchhammer, Heike Brötz-Oesterhelt, Boris Macek, Silja Mordhorst, Leonard Kaysser, Bertolt Gust

**Affiliations:** 1 Pharmaceutical Biology, Pharmaceutical Institute, Eberhard-Karls-University Tübingen 493697 , Tübingen, Germany; 2 Partner Site Tübingen, German Centre for Infection Research (DZIF) 459706, Tübingen, Germany; 3 Department of Microbial Bioactive Compounds, Interfaculty Institute of Microbiology and Infection Medicine, Eberhard-Karls-University Tübingen, Tübingen, Germany; 4 Department of Microbiology and Organismic Interactions, Interfaculty Institute of Microbiology and Infection Medicine, Eberhard-Karls-University Tübingen, Tübingen, Germany; 5 Proteome Center Tübingen, Institute of Cell Biology, Eberhard-Karls-University Tübingen, Tübingen, Germany; 6 Interfaculty Institute for Bioinformatics and Medical Informatics, Eberhard-Karls-University Tübingen, Tübingen, Germany; 7 Cluster of Excellence Controlling Microbes to Fight Infections, Eberhard-Karls-University Tübingen 9188, Tübingen, Germany; 8 Institute for Drug Discovery, Department of Pharmaceutical Biology, Leipzig University 9180, Leipzig, Germany; Centers for Disease Control and Prevention, Atlanta, Georgia, USA

**Keywords:** TetR regulator, Crp regulator, heterologous expression, *Streptomyces*, regulation of heterologously expressed antibiotic gene clusters, liponucleoside antibiotics

## Abstract

Heterologous expression in well-studied model strains is a routinely applied method to investigate biosynthetic pathways. Here, we pursue a comparative approach of large-scale DNA-affinity-capturing assays (DACAs) coupled with semi-quantitative mass spectrometry (MS) to identify putative regulatory proteins from *Streptomyces coelicolor* M512, which bind to the heterologously expressed biosynthetic gene clusters (BGCs) of the liponucleoside antibiotics caprazamycin and liposidomycin. Both gene clusters share an almost identical genetic arrangement, including the location of promoter regions, as detected by RNA sequencing. A total of 2,214 proteins were trapped at the predicted promoter regions, with only three binding to corresponding promoters in both gene clusters. Among these, the overexpression of a yet uncharacterized TetR-family regulator (TFR), Sco4385, increased caprazamycin but not liposidomycin production. Protein–DNA interaction experiments using biolayer interferometry confirmed the binding of Sco4385 to P*cpz10* and P*lpmH* at different locations within both promoter regions, which might explain its functional variance. Sequence alignment allowed the determination of a consensus sequence present in both promoter regions, to which Sco4385 was experimentally shown to bind. Furthermore, we found that the overexpression of the Crp regulator, Sco3571, leads to a threefold increase in caprazamycin and liposidomycin production yields, possibly due to an increased expression of a precursor pathway.

**IMPORTANCE:**

Streptomycetes are well-studied model organisms for the biosynthesis of pharmaceutically, industrially, and biotechnologically valuable metabolites. Their naturally broad repertoire of natural products can be further exploited by heterologous expression of biosynthetic gene clusters (BGCs) in non-native host strains. This approach forces the host to adapt to a new regulatory and metabolic environment. In our study, we demonstrate that a host regulator not only interacts with newly incorporated gene clusters but also regulates precursor supply for the produced compounds. We present a comprehensive study of regulatory proteins that interact with two genetically similar gene clusters for the biosynthesis of liponucleoside antibiotics. Thereby, we identified regulators of the heterologous host that influence the production of the corresponding antibiotic. Surprisingly, the regulatory interaction is highly specific for each biosynthetic gene cluster, even though they encode largely structurally similar metabolites.

## INTRODUCTION

**Fig 1:**
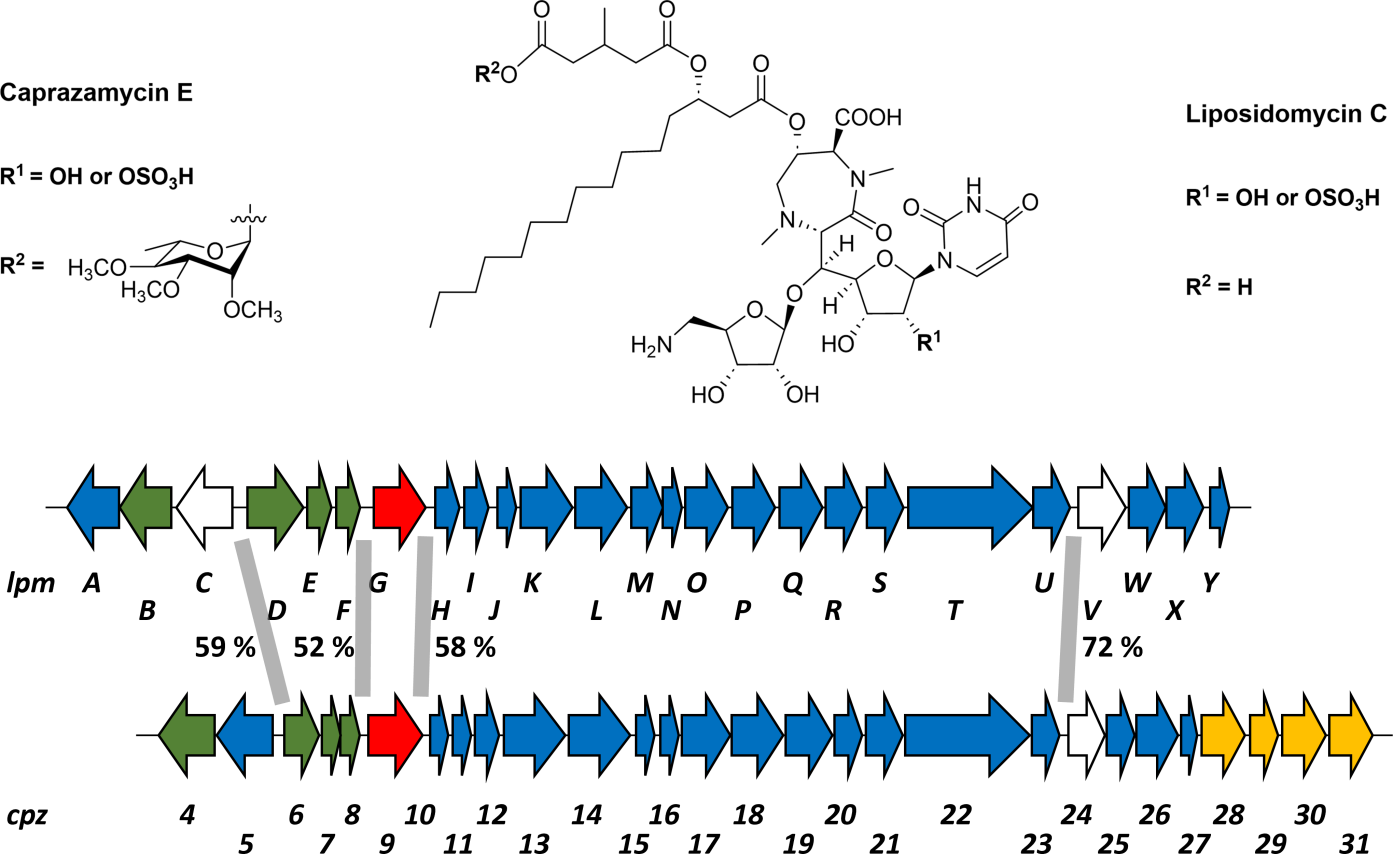
Comparison of caprazamycins and liposidomycins (top). Commonalities and differences in the chemical structure of caprazamycin E and liposidomycin C, shown as representatives for these two liponucleoside antibiotics. Because the heterologous host, *S. coelicolor*, is lacking genes for the biosynthesis of the rhamnosyl moiety at R^2^, heterologous expression of the caprazamycin gene cluster resulted in caprazamycin aglyca instead of caprazamycin formation (bottom). Comparison of the genetic arrangement of the caprazamycin and liposidomycin BGC. Corresponding promoter pairs (assigned as P*cpz6*/P*lpmD*, P*cpz9*/P*lpmG*, P*cpz10*/P*lpmH*, and P*cpz24*/P*lpmV*) of both clusters that were applied in the DACAs are marked with gray bars, and their DNA sequence identity is indicated in percentages. Color code for gene functions: biosynthesis and transfer of the sulfate group (green); unknown function (white); cluster-situated regulators (red); biosynthesis, resistance, and transport (blue); and transfer and methylation of the rhamnosyl moiety (yellow).

Caprazamycins are liponucleoside antibiotics naturally produced by *Streptomyces* sp. MK730-62F2 and were discovered in 2003 (1). These compounds consist of a central (+)-caprazol ring structure with an attached β-hydroxy fatty acid of variable length, by which we differentiate between caprazamycins A-G. The β-hydroxy fatty acid is linked to an uncommon 3-methylglutaryl (3-MG) moiety (Fig. 1). Caprazamycins exhibit strong antimycobacterial activity, for example, against a range of drug-resistant strains of *Mycobacterium smegmatis* (2) and clinically relevant strains of *Mycobacterium tuberculosis* at minimum inhibitory concentrations (MICs) of 6.25–12.5 µg/mL (1). Semi-synthetic analogs of caprazamycin were generated, and among them, compound CPZEN-45, a 4-butylanilide derivative, showed the highest antibacterial activity and improved pharmacological properties (3). This compound entered clinical trials for the treatment of tuberculosis and is currently tested as a drug candidate in aerosol powder form for inhaled administration (4). The mode of action of caprazamycins is to inhibit the phospho-MurNAc-pentapeptide translocase I (MraY translocase), which catalyzes the first membrane-bound step in bacterial cell wall biosynthesis (5, 6). We previously identified the biosynthetic gene clusters (BGCs) to produce caprazamycins and the closely related liposidomycins in *Streptomyces* sp. MK730-62F2 and *Streptomyces* sp. SN-1061M, respectively (7, 8). For further investigation, both gene clusters were heterologously expressed in *Streptomyces coelicolor* M512, generating strains *S. coelicolor* M512/cpzLK09 and *S. coelicolor* M512/lpmLK01. Liposidomycins carry a characteristic sulfate group at the (+)-caprazol, but sulfated caprazamycins have also been detected to a smaller extent (9). Caprazamycins feature a permethylated rhamnose moiety attached to the 3-MG motif. However, as the genes for the synthesis of the deoxysugar are encoded outside of the gene cluster, only aglycons are produced upon heterologous expression (10). The formation of liponucleoside antibiotics, especially of caprazamycins, has been extensively studied over the past decade (11–14). The shared structural and biosynthetic features of caprazamycins and liposidomycins strongly indicate matching dependencies on primary metabolism for precursor supply. Interestingly, both BGCs show a highly similar genetic arrangement, and putative promoter regions are located at the same positions with regard to their genetic neighborhood (referred to as corresponding promoter regions). All four pairs of corresponding promoter regions share a high degree of DNA sequence identity, ranging between 52% and 72% (Fig. 1).

Cluster-situated regulation of the caprazamycin and liposidomycin BGCs is likely facilitated by the homologous AraC-type regulators Cpz9 and LpmG, respectively, which exhibit 77% sequence similarity on the protein level.

These structural and genetic homologies raise the question of whether the similarities extend to the regulatory networks both pathways are embedded in. Especially as the biosynthesis of caprazamycins and liposidomycins would feed from the same routes of primary metabolism. This is of particular relevance when a biosynthetic pathway is transferred between bacteria, e.g., by horizontal gene transfer or by the introduction into a heterologous host.

We previously investigated the regulatory influence of *S. coelicolor* M512 on the heterologously expressed gene cluster for the biosynthesis of the aminocoumarin antibiotic novobiocin. This study revealed a “crosstalk” between the host primary metabolism and the novobiocin BGC, mediated by regulators that directly bind to the heterologous gene cluster and modulate novobiocin precursor supply as well (15, 16). Therefore, we used DNA-affinity-capturing assays (DACAs) with subsequent semi-quantitative mass spectrometry (MS) as a powerful tool to screen the whole proteome of a bacterial culture at a fixed time point for binding to an immobilized DNA sequence. This method has also been applied to investigate the regulation of the antibiotic gene clusters encoding for actinorhodin and undecylprodigiosin (17).

We now demonstrate that fine-tuned regulation of the heterologous host *S. coelicolor* M512 affects the expression of two similar biosynthetic pathways, thereby manipulating the production of caprazamycins and liposidomycins. Using comparative DACAs, we show that regulatory proteins from the host indeed bind to the heterologously expressed gene clusters and affect the production of the respective compounds. We identify the binding site of an uncharacterized TetR-family regulator (TFR) and present a deduced consensus sequence for this protein.

## RESULTS

### Identification of putative promoter regions and transcriptome analysis of the heterologous producer strains

A comparison of the genetic organization of the caprazamycin and liposidomycin BGC shows obvious similarities (Fig. 1). All genes are arranged in the same order, with most of them sharing significant sequence similarity with each other, indicating common mechanisms for biosynthesis, transport, and regulation. The highly conserved AraC-type cluster-situated regulators (CSRs), Cpz9 and LpmG, exhibit 77% similarity (69% identity) on amino acid level. Although both CSRs affected caprazamycin and liposidomycin production, respectively, only LpmG overexpression resulted in a significant threefold increase in liposidomycin production (Fig. S1). This is in accordance with previous reports that AraC-type regulators typically function as transcriptional activators (18). Notably, *cpz9* overexpression did not have an effect on caprazamycin production. We thus speculate that the bottleneck in this pathway is not related to the abundance of its biosynthetic enzymes. To identify regions in the BGCs that could be targeted by regulatory proteins from *S. coelicolor* M512, we searched for intergenic regions larger than 100 bp. We determined four putative promoter sites, which are located at the same positions within both clusters. These corresponding promoter pairs were designated as P*cpz6*/P*lpmD*, P*cpz9*/P*lpmG*, P*cpz10*/P*lpmH*, and P*cpz24*/P*lpmV*, which share 59%, 52%, 58%, and 72% of DNA sequence identity, respectively (Fig. 1). The intergenic region of 302 bp between *cpz5* and *cpz6* (431 bp between *lpmC* and *lpmD*) most likely contains promoters for both genes, which are transcribed in opposite directions. Another putative promoter region was identified upstream of the regulatory genes *cpz9* and *lpmG* (103 and 259 bp, respectively). Overlapping open reading frames and intergenic regions <90 bp indicate that the genes *cpz10-cpz31*/*lpmH-lpmY* are likely transcribed as one polycistronic mRNA. This makes the putative promoters of *cpz10* and *lpmH* (149 and 128 bp, respectively) an essential regulatory node. The last selected pair of intergenic regions, P*cpz24* (280 bp) and P*lpmV* (286 bp), would be large enough to facilitate a potential binding of the RNA polymerase and was therefore included in the DACA experiments.

The putative promoter regions were selected as bait sequences for DACAs in order to identify binding proteins from the heterologous host *S. coelicolor* M512. Confirmation of promoter regions was obtained by RNA sequencing of three biological replicates of each heterologous producer strain (*S. coelicolor* M512/cpzLK09 and *S. coelicolor* M512/lpmLK01). Analysis of the transcriptome data revealed a highly similar operon structure for the caprazamycin and liposidomycin BGC with a continuous transcription of genes *cpz10-cpz31* and *lpmH-lpmY,* respectively. However, there is a slight increase in transcription of genes *cpz24* and *lpmV*. As the intergenic regions upstream of these genes are large enough to serve as putative promoters, we decided to include these regions in the following DACA experiments, although their transcriptional impact seemed to be of minor importance. As regions P*cpz6* and P*lpmD* form a bidirectional promoter and neighboring genes *cpz5*/*cpz6* and *lpmC*/*lpmD* show strong transcription in our transcriptome data, these regions were included as well. We were also interested in investigating which regulators from the heterologous host bind directly upstream of the CSR genes. Therefore, we included regions P*cpz9*/P*lpmG*. According to the RNA-sequencing results, the putative promoter pair of P*cpz10*/P*lpmH* confers the strongest transcriptional activity within both gene clusters (Fig. S2 to S5). These two regions were thus included as bait sequences for the DACAs as well. For amplification, all intergenic regions were extended, reaching into the neighboring genes to ensure the trapping of proteins whose binding sites may overlap with the codon regions.

We additionally screened the caprazamycin and liposidomycin BGCs for transcription factor binding sites *in silico*, using the recently published database LogoMotif (19). However, only one potential binding site could be detected in each BGC, and no regulator could be predicted to bind to both gene clusters. Therefore, we pursued our goal to identify binding proteins by experimental approaches using DACAs.

### 20 L cultivation of heterologous strains for the production of whole-cell protein

**Fig 2:**
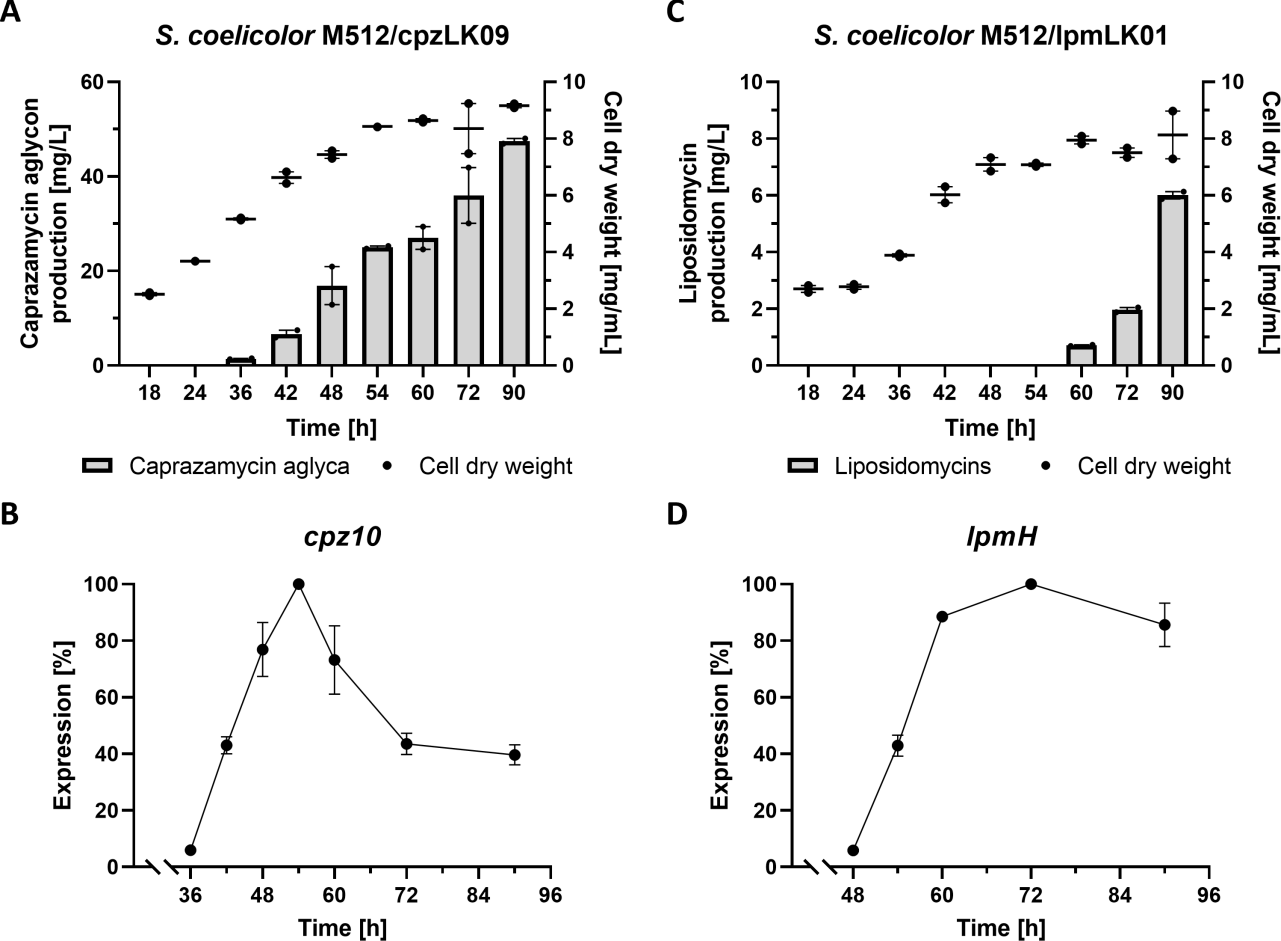
Growth curve and production levels of *S. coelicolor* M512/cpzLK09 (A) and *S. coelicolor* M512/lpmLK01 (C) during 20 L fermentation. RT-qPCR revealed transcription rates of the first biosynthetic genes, *cpz10* (B) and *lpmH* (D), of the corresponding operon. Each bar represents the mean production measured in technical duplicate, and the error bars represent the range. The highest transcription was set to 100%.

To obtain whole-cell protein for the DACA, *S. coelicolor* M512/cpzLK09 and *S. coelicolor* M512/lpmLK01 were both individually cultivated in 20 L bioreactors. Samples for the determination of cell dry weight and liponucleoside production, as well as samples for RNA and protein extraction, were collected over a period of 90 hours (Fig. 2). In this experimental setup, *S. coelicolor* M512/cpzLK09 entered the exponential growth phase 18 hours after inoculation. Caprazamycin aglycon levels constantly increased over 90 hours up to approximately 50 mg/L. *S. coelicolor* M512/lpmLK01 exhibited a lag phase for 36 hours after inoculation and then entered exponential growth. Liposidomycin production was detectable only after 60 hours, but levels increased rapidly up to 6 mg/L after 90 hours of fermentation (Fig. 2A and C). Transcription rates of the biosynthetic genes *cpz10* and *lpmH* were lowest after 36 and 48 hours, and highest after 54 and 72 hours, respectively (Fig. 2B and D). Therefore, protein samples from *S. coelicolor* M512/cpzLK09 at 36 and 54 hours and samples from *S. coelicolor* M512/lpmLK01 at 48 and 72 hours were applied for the DACA. The two selected time points reflect different stages of cell growth, either before or at the time of maximum production of liponucleoside antibiotics. This enhances the probability of trapping both activators and repressors that may only be expressed over limited periods of time.

### Identification of binding proteins using DACA

Intergenic regions, flanked by sections of the down- and upstream genes, were amplified and biotinylated by PCR, resulting in DNA fragments of 350–560 bp in length. The extended promoter region of the vegetative sigma factor gene *hrdB* (P*hrdB*) was included as a negative control. The immobilized promoter regions from the caprazamycin or liposidomycin BGC were exposed to whole-cell protein extract from *S. coelicolor* M512/cpzLK09 and *S. coelicolor* M512/lpmLK01, respectively. A total of 2,214 proteins were trapped on the promoter regions and identified by subsequent mass spectrometry with semi-quantitative abundances measured as label-free quantification (LFQ) intensities between 1.67E+07 and 1.2E+12 (see Data Set S1 for a full list of all detected proteins). The vast majority of them bound to more than one tested promoter (83% and 85% in the DACAs with the caprazamycin and liposidomycin promoter regions, respectively) and were considered nonspecific. At the four intergenic regions of the caprazamycin BGC (and the negative control P*hrdB*), a total of 1,906 proteins were detected, while at the liposidomycin BGC, 1,540 proteins were detected. Comparing both data sets, most proteins bound at least once in both gene clusters (1,232), 674 proteins were found to bind exclusively in the caprazamycin BGC and 308 in the liposidomycin BGC. By defining specific binding as binding to only one of the promoter regions of both gene clusters, we drastically reduced the number of proteins of interest (Table 1). However, regarding this strict definition of specificity, it must be noted that even proteins binding to multiple promoters within one gene cluster (but not P*hrdB*) may still represent specific binders. For example, the putative cluster-situated regulator of the caprazamycin gene cluster, Cpz9, was found to bind to all tested promoter regions of its cluster.

**TABLE 1:** Numbers of proteins binding in the DACA^*a*^

*S. coelicolor* M512/cpzLK09			*S. coelicolor* M512/lpmLK01		
Total proteins	1,906	(234)	Total proteins	1,540	(220)
Unspecific	1,575	(182)	Unspecific	1,305	(161)
Specific for:			Specific for:		
P*cpz6*	32	(9)	P*lpmD*	20	(9)
P*cpz9*	59	(13)	P*lpmG*	22	(12)
P*cpz10*	58	(14)	P*lpmH*	31	(19)
P*cpz24*	113	(8)	P*lpmV*	15	(7)
P*hrdB*	69	(8)	P*hrdB*	147	(12)

^*a*^ Specific binding is defined as binding solely to one promoter region in both clusters. Numbers in parentheses indicate numbers of proteins annotated as possible regulators.

Our main aim in this study was to compare the regulation between the heterologously expressed caprazamycin and liposidomycin BGCs. Therefore, we used the binding of the corresponding promoter pairs (Fig. 1) as the main criterion for protein candidate identification. We searched for proteins that only bound to either one of the corresponding promoter pairs (P*cpz6*/P*lpmD*, P*cpz9*/P*lpmG*, P*cpz10*/P*lpmH*, or P*cpz24*/P*lpmV*) and ended up with three candidates matching this criterion (Table S6). These were a MarR-type regulator (Sco2987), which bound to P*cpz6* and P*lpmD*, and two TetR-family regulators (Sco4385 and Sco5956), binding to the P*cpz10*/P*lpmH* promoter pair. For the caprazamycin BGC, Sco2987 was found only at 54 hours, while for the liposidomycin gene cluster, it was detected at both tested time points. LFQ intensities were roughly similar in all samples in which Sco2987 was found, ranging from 1.35E+08 to 7.52E+08. The TFR Sco4385 was detected at more than 10-fold higher rates on the P*lpmH* region (average LFQ intensities of both time points were 5.22E+09) than on the *cpz10* promoter region (average LFQ intensities of both time points were 3.18E+08). On both promoters, protein intensities were higher at the first time point (Table S6). Sco5956 seems to be expressed primarily during the beginning of liponucleoside production, as it was only detected in the samples of early time points of growth (36 and 48 hours). LFQ intensities of this protein were similar on both P*cpz10* and P*lpmH* with values of 1.02E+08 and 2.39E+08, respectively.

As expected, some well-known global *Streptomyces* regulators such as AdpA, NdgR, SlbR AtrA, and Rok7B7 were also trapped nonspecifically on the tested promoter regions. These proteins were found on average at very high LFQ intensities with values up to 3.42E+11 (AdpA), emphasizing their importance as major cellular regulators (Table S6).

In addition to selecting protein candidates based on promoter binding in the DACAs, we performed a literature search for known regulators that are involved in modulating the precursor supply of caprazamycins and liposidomycins. One precursor supply pathway, which was recently studied by our group, is the leucine-isovalerate-utilization (*liu*-) pathway, comprising genes *sco2776-sco2779* in *S. coelicolor*. Bär et al. (20) showed that the 3-methylglutaryl moiety, incorporated in caprazamycin aglyca and liposidomycins, partly originates from the *liu*-pathway via 3-methylglutaconyl-CoA as a highly unusual precursor in natural product biosynthesis. Interestingly, a well-studied cAMP receptor protein (Crp)-type regulator, Sco3571, was shown to strongly activate the transcription of the first *liu*-pathway gene *sco2776* by Gao et al. (21). The reported putative binding motif for Sco3571 is also present in the corresponding promoter regions of P*cpz6*/P*lpmD* and P*cpz9*/P*lpmG*. Gratifyingly, Sco3571 was readily found among the proteins binding to the promoter regions used for the DACA experiments. However, it bound nonspecifically to all tested promoter regions *in vitro* (Table S6). Although Sco3571 did not match our selection criteria, we included this protein in our study due to its known involvement in regulating precursor supply and the presence of its binding site in both heterologously expressed gene clusters.

### Overexpression of *sco2987, sco3571, sco4385*, and *sco5956* and deletion of selected candidates

**Fig 3:**
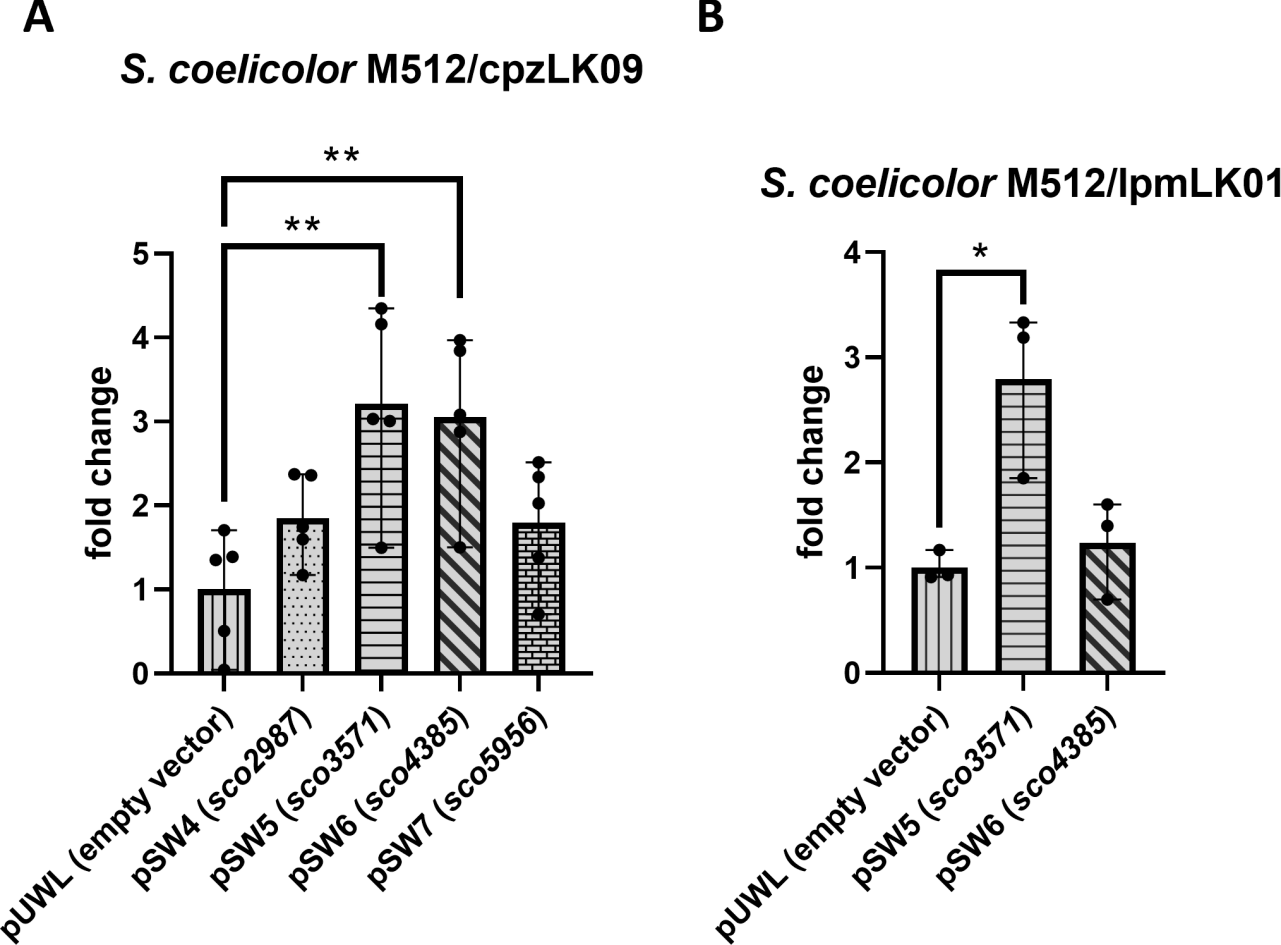
Liponucleoside production of *S. coelicolor* M512/cpzLK09 (A) and *S. coelicolor* M512/lpmLK01 (B) overexpressing candidate regulatory genes identified in the DACAs. Production levels are correlated to the empty vector control (pUWL-apra-oriT). Each bar represents the mean, and the error bars represent the range of production of independent replicates. Statistically significant differences were tested using unpaired two-tailed *t*-tests, where * signifies a *P* value < 0.05 and ** a *P* value < 0.01.

Because results from the DACAs are derived by *in vitro* experiments and were designed to detect potentially interesting candidates for further investigation, we now aimed to analyze the selected candidates *in vivo*. Thus, the role of the putative regulators Sco2987, Sco4385, and Sco5956, binding to corresponding promoters of the caprazamycin and liposidomycin BGC, was investigated for their impact on caprazamycin production. To this end, the genes *sco2987*, *sco4385*, and *sco5956* were individually cloned under the control of the strong, constitutive ermE* promoter into the replicative vector pUWL-apra-oriT, resulting in plasmids pSW4 (*sco2987*), pSW6 (*sco4385*), and pSW7 (*sco5956*). The constructs were introduced into *S. coelicolor* M512/cpzLK09 by conjugation, using the non-DNA-methylating *Escherichia coli* strain ET12567 as a shuttle. Cultivation of the resulting mutants and subsequent extraction of liponucleosides from the spent medium revealed a significant increase in caprazamycin production in the mutant overexpressing *sco4385* (Fig. 3A). The production level in this strain was three times higher than in the control strain harboring the empty vector. The other tested regulators, Sco2987 and Sco5956, showed no significant effect on caprazamycin production *in vivo*.

The same procedure was applied for gene *sco3571*, encoding the Crp-type regulator known to be involved in the regulation of caprazamycin precursor supply. Cloning of the gene into the replicative vector pUWL-apra-oriT generated plasmid pSW5. Cultivation of *S. coelicolor* M512/cpzLK09 carrying pSW5 resulted in a significant increase of caprazamycin aglycon levels by about three-fold (Fig. 3A).

Because the overexpression of genes *sco4385* and *sco3571* showed an impact on caprazamycin aglycon production *in vivo*, we continued our study with these two candidates. Therefore, pSW5 (encoding Crp-type regulator Sco3571) and pSW6 (encoding TetR-type regulator Sco4385) were transferred into *S. coelicolor* M512/lpmLK01. Again, the presence of pSW5 elevated production levels by approximately threefold. Interestingly, under the influence of pSW6, the production levels were within the boundaries of biological variability as in the empty vector control (Fig. 3B). The enhanced liponucleoside production by *sco3571* overexpression may be explained by an improved supply of the 3-MG precursor via the *liu*-pathway (20). The regulatory mode of action of Sco4385 is unknown, but the different effects of its overexpression within the two heterologous producer strains spiked our interest. To identify the underlying molecular mechanisms of Sco3571 and Sco4385, we next aimed to perform gene deletion studies. Thus, we deleted both genes individually in the heterologous strains *S. coelicolor* M512/cpzLK09 and *S. coelicolor* M512/lpmLK01. While the deletion of gene *sco3571* led to accelerated growth and sporulation of the mutant strains, in accordance with previous observations (21), the deletion of *sco4385* showed no morphological phenotype. Cultivation and semi-quantitative liquid chromatography-mass spectrometry (LC-MS) analysis of the culture extracts revealed that the deletion of s*co3571* in the heterologous caprazamycin producer strain led to a reduction in production rates of 15%–53%, compared to the intact strain. This result supports the hypothesis that Sco3571 is a positive regulator of heterologous caprazamycin production. However, the results for *S. coelicolor* M512/lpmLK01 were inconclusive in all repeated cultivations of the *sco3571* knockout mutants. The individual mutants showed considerable variations in liposidomycin production of 70%–640%, compared to the unmodified heterologous strain. The deletion of gene *sco4385* had no significant impact on antibiotic production in both heterologous producer strains (data not shown).

Hence, we speculated that the recognition sites for Sco4385 within the promoters P*cpz10* and P*lpmH* could be located at different positions, allowing the regulator to facilitate diverging effects on the downstream genes upon overexpression. To test this hypothesis, we intended to determine the binding sites of Sco4385 within P*cpz10* and P*lpmH*.

### Determination of the Sco4385 binding site within P*cpz10* and P*lpmH*

**Fig 4:**
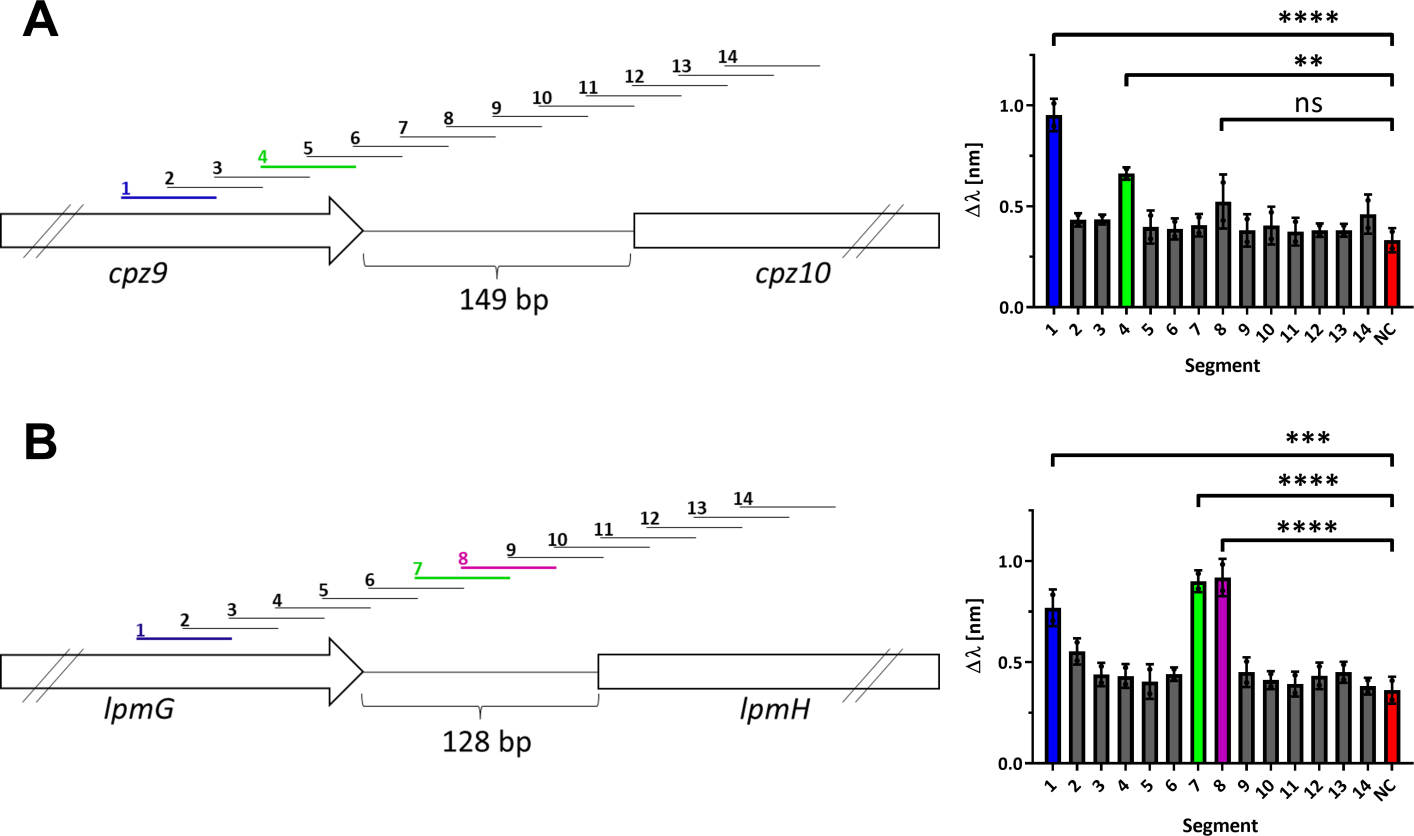
Binding of Sco4385 to segments covering the extended promoter regions of P*cpz10* (A) and P*lpmH* (B). The extended promoter regions were divided into 14 segments of 50 bp length and were tested for Sco4385 binding by biolayer interferometry. (A) Localization of the segments in the extended P*cpz10* region (left) and intensity of the wavelength shift induced by the binding of Sco4385 at these segments (right). Bars and error bars represent the mean and the standard deviation of two replicates. Asterisks (**, ****) indicate significant differences to the negative control (NC), calculated using one-way ANOVA *P* < 0.0001, α = 0.05. (B) Localization of the segments in the extended P*lpmH* region (left) and intensity of the wavelength shift induced by the binding of Sco4385 at these segments (right). Bars and error bars represent the mean and the standard deviation of two replicates. Asterisks (***, ****) indicate significant differences to the negative control (NC), calculated using one-way ANOVA *P* < 0.0001, α = 0.05.

To investigate the differential role played by Sco4385 in caprazamycin and liposidomycin biosynthesis upon overexpression, we employed *in vitro* binding assays supported by biolayer interferometry (BLI). The His-tagged protein (24.69 kDa) was overproduced in *E. coli* BL21 DE3 and was purified via Ni-NTA chromatography and size exclusion chromatography (Fig. S6). Investigation of Sco4385 via size exclusion chromatography with multi-angle light scattering (SEC-MALS) revealed a dimeric state of the protein (Fig. S7). The extended promoter regions of *cpz10* and *lpmH*, as applied in the DACA experiments, were divided into 14 segments, respectively. Thus, each segment spans 50 bp in length and overlaps with the upstream and downstream segments by 25 bp (Fig. 4). Incorporation of a biotin tag was performed through the annealing of complementary oligonucleotides with the anti-sense strand carrying a single-stranded overhang for a biotinylated ReDCaT linker (22). DNA–protein interaction of Sco4385 with the 50 bp DNA segments was assessed using BLI. This technique detects molecular binding to a coated sensor surface through the wavelength shift of a light beam, which is proportional to the thickness and molecular weight bound to the surface (23). A control experiment with unspecific DNA (50 bp segment of the *hrdB* promoter) was also conducted as a negative control. This resulted in a minor wavelength shift of 0.35 ± 0.05 nm. The analysis of all recorded shifts (Fig. S8 and S9) revealed distinctive binding at P*cpz10* 1 and P*cpz10* 4 with intensities of 1.01 ± 0.06 nm and 0.66 ± 0.02 nm, respectively (Fig. 4A). The most prominent binding in the *lpmH* promoter is observed in segments 1, 7, and 8 (Fig. 4B). With 0.91 ± 0.04 nm and 0.92 ± 0.06 nm, segments 7 and 8 of P*lpmH* are bound by Sco4385 with similar intensities. The differential dissociation pattern of Sco4385 with segments 7 and 8 indicates that the binding position is not entirely covered by the overlapping region alone (Fig. S9). The binding at segment 1 with 0.77 ± 0.06 nm showed a slightly reduced intensity compared to segments 7 and 8 (Fig. 4B). This finding validates the results from the DACA that Sco4385 binds to both extended promoter regions, P*cpz10* and P*lpmH*.

P*cpz10* segments 1 and 4 are located within the coding region of *cpz9*, ending 75 bp upstream and directly at its stop codon, respectively. Notably, this leaves the actual intergenic region between *cpz9* and *cpz10* available for binding of other potential activators/regulators, e.g., Cpz9. In P*lpmH,* Sco4385 binds within the coding region of the corresponding gene *lpmG* in segment 1 of P*lpmH*, which ends 62 bp upstream of the stop codon. However, Sco4385 additionally binds segments 7 and 8, situated in the middle of the intergenic region. In contrast to the binding of P*cpz10*, the binding of Sco4385 in P*lpmH* may act as a blockade to other potential regulators in this region like the cluster-situated regulator LpmG.

**Fig 5:**
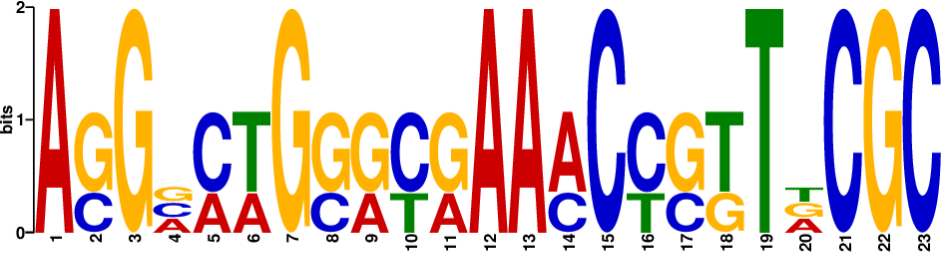
A 23 bp consensus sequence, based on the oligos that were bound by Sco4385 in the BLI measurements, was determined using the software GLAM2 (24).

In the next step, we screened these sequences for common motifs serving as potential recognition sites for Sco4385. Using the online tool gapped local alignment of motifs (GLAM2; [24]), we discovered a 23 bp consensus sequence (Fig. 5) being present in segment 1 of P*cpz10*, segment 1 of P*lpmH*, and the overlapping region of segments 7 and 8 of P*lpmH* (Fig. S12). These 23 bp sequences were designated as P*cpz10*_1_23 bp, P*lpmH_*1_23 bp, and P*lpmH_*7/8_23 bp, respectively. While P*cpz10*_1_23 bp and P*lpmH_*1_23 bp are highly similar (83% DNA identity), P*lpmH_*7/8_23 bp shares only 43% DNA identity with the other two putative recognition sequences.

With the predicted Sco4385 binding sites at hand, we re-analyzed our RNA-sequencing data for the transcription start sites (TSSs) in P*cpz10* and P*lpmH*. The regulator binding site in P*cpz10* is far upstream of the predicted TSS, making interference between regulator binding and transcription unlikely. In contrast, in the liposidomycin gene cluster, the Sco4385 binding site is in the center of P*lpmH* and only about 20 bp downstream of the TSS. Therefore, Sco4385 binding may interfere with the transcriptional machinery at this position.

For the experimental validation of P*cpz10*_1_23 bp, P*lpmH_*1_23 bp, and P*lpmH_*7/8_23 bp as recognition sequences, we tested Sco4385 binding to the respective double-stranded oligonucleotides by BLI. Indeed, the binding of P*cpz10*_1_23 bp and P*lpmH_*1_23 bp was consistent with the binding to the whole segments, confirming this sequence to be sufficient for Sco4385 recognition and binding. In the case of P*lpmH_*7/8_23 bp, the binding of Sco4385 dropped severely in comparison to segments 7 and 8 of P*lpmH*, indicating that at this position, the recognition sequence was not entirely covered by the 23 bp sequence and may be marginally offset (Fig. S10).

## DISCUSSION

Heterologous expression of BGCs is routinely applied for various purposes. Some bacterial strains are optimized to minimize the production of secondary metabolites from the host itself (25), thereby aiming at an enhanced production of the desired compound with less metabolic background. It is also a successfully employed approach to circumvent working with pathogens, such as biosafety level 2 organisms (26), and to facilitate genetic studies on the respective BGC (27). At the same time, the regulatory fine-tuning of the BGC or metabolic changes occurring within the host organism are largely underexplored. Here, we addressed this question by using the highly similar caprazamycin and liposidomycin gene cluster for a comparative approach to unravel interactions between the host’s proteome and the newly incorporated BGCs.

With our approach using DACAs and subsequent protein detection via mass spectrometry, we identified a new regulator influencing caprazamycin production. By focusing on proteins that bind solely to corresponding promoters of both gene clusters, we identified three proteins out of 2,214 host proteins, matching this criterion. A comparably low number in contrast to the overwhelming amount of binding proteins initially identified in our assay. This could indicate that the regulation of the caprazamycin and liposidomycin BGCs in the same heterologous host is rather BGC specific and distinct, even for the biosynthesis of these two highly similar compounds. The identified TFR Sco4385 bound specifically to the predicted main promoter regions of both gene clusters, P*cpz10* and P*lpmH*. Based on our RNA-sequencing data, these promoter regions confer a strong impact on the transcription of downstream biosynthetic genes (Fig. S2 to S5). This observation was further supported as overexpression of *sco4385* in the heterologous caprazamycin producer strain significantly increased caprazamycin production. However, overexpression in the heterologous liposidomycin producer strain did not affect liposidomycin production levels. Notably, TFRs are the most abundant group of transcriptional regulators in *Streptomyces* (28). The genetic organization of *sco4385* and neighboring genes suggests a putative gene cluster ranging from *sco4383* to *sco4386* (Fig. S11). While the function of gene *sco4386* remains unknown, gene *sco4383* is annotated as a 4-coumarate-coenzyme A (CoA) ligase and *sco4384* as an enoyl-CoA hydratase. These enzymes are most likely involved in fatty acid metabolism. Although the majority of TFRs act as transcriptional repressors, there are numerous examples of TetR regulators operating as activators (29–31) or as proteins with dual regulatory functions (32). Their highly conserved structure consists of nine α-helices, of which helices one to three are involved in DNA binding, while helices four to nine form a ligand-binding domain (33). This characteristic tertiary structure perfectly matches the protein structure of Sco4385 as calculated by Colabfold (34), supporting its classification to the TetR regulator family. Based on our SEC-MALS measurements, Sco4385 is present in a homodimeric state as it is the case for most TFRs. Nevertheless, a common binding sequence for TFRs does not exist due to their highly variable DNA-binding domain. Aiming for a hypothesis to explain the differential impact of Sco4385 on caprazamycin and liposidomycin production, we used BLI assays to validate Sco4385 binding to P*cpz10* and P*lpmH*. Thereby, we revealed a binding site in the regions encoding for the cluster-situated regulators, Cpz9 and LpmG, respectively, which were part of the extended promoter regions. An additional binding site in P*lpmH* in the center of the intergenic region between *lpmG* and *lpmH* was identified. The presence of binding sites at different positions within P*cpz10* and P*lpmH* might explain differential effects concerning the production yields for caprazamycins and liposidomycins. This observation demonstrates that the same regulatory protein may confer different outcomes in the same host strain expressing highly similar BGCs. Our results therefore imply that the regulation of every heterologously expressed gene cluster needs to be investigated individually.

We also applied the deduced consensus binding sequence for Sco4385 in a whole genome search in *S. coelicolor*, but no alternative binding sites were identified. Only when allowing two mismatches was the sequence found at six positions within the genome. However, all matches are localized within coding regions of genes, stressing the fact that the binding sites within the heterologously expressed gene clusters may not represent their genuine recognition sequence.

Besides the investigation of new regulators, such as Sco4385, we researched literature for known regulators with an involvement in precursor supply. Thereby, we identified Sco3571 from the *S. coelicolor* M512 host, which regulates a precursor pathway of caprazamycin and liposidomycin biosynthesis. Its published binding site (21) is present in two of the corresponding promoter pairs (P*cpz6*/P*lpmD* and P*cpz10* and P*lpmH*). However, in our DACAs, Sco3571 was trapped on all our tested promoter regions in both the caprazamycin and liposidomycin gene cluster. According to Gao et al. (21), overexpression of *sco3571* by an inducible promoter enhances transcription of *sco2776* (*liuB*), the first gene of the *liu*-pathway, by approximately 17-fold. Bär et al. (20) recently described how the central intermediate in 3-MG biosynthesis, 3-methylglutacoyl-CoA, can originate from two different biosynthetic routes, i.e., via the host’s *liu* pathway and via the cluster-situated 3-hydroxy-3-methylglutaryl-CoA synthase Cpz5. Therefore, the stronger transcription of the *liu*-pathway could, to some extent, be the reason for increased caprazamycin production yields, which we observed when overexpressing *sco3571* in the heterologous producer strain. As the 3-MG moiety is a central building block in liposidomycin biosynthesis as well, the same mechanism most probably applies to the enhanced production yields of *S. coelicolor* M512/lpmLK01 upon *sco3571* overexpression. The overexpression of *sco3571* in both heterologous strains, *S. coelicolor* M512/cpzLK09 and *S. coelicolor* M512/lpmLK01, strongly increased the production of the respective liponucleoside. This demonstrates that DACA studies can be applied to screen for regulators with known or unknown function. Such proteins are promising candidates in strain optimization strategies to enhance the production of a desired natural product.

While it is known that Sco3571 is a highly conserved and widely distributed regulator in Streptomycetes (21), the regulatory significance of Sco4385 is yet unknown. A BLAST analysis revealed that it is also highly conserved and widely distributed, with over 100 different *Streptomyces* strains carrying homologs with over 90% identity to Sco4385 on the amino acid level. Furthermore, other *Streptomyces* strains commonly used as heterologous hosts, such as *Streptomyces lividans*, *Streptomyces griseus*, and *Streptomyces albus*, contain homologs with high similarity as well (99%, 86%, and 83%, respectively).

Moreover, the regulatory impact of the host-encoded regulators Sco3571 and Sco4385 on heterologous liponucleoside biosynthesis raised the question of whether similar regulatory mechanisms could apply to the native producer strains as well. Therefore, we searched for orthologs in *Streptomyces* sp. MK730-62F2 and *Streptomyces* sp. SN-1061M, from which the caprazamycin and liposidomycin BGCs were derived, respectively (7, 8). For the Crp regulator Sco3571, we identified orthologs in both strains exhibiting 97%–98% identity on amino acid level, respectively. Furthermore, we identified orthologs of Sco4385, showing 87% identity (90% similarity) in *Streptomyces* sp. MK730-62F2 and 86% identity (90% similarity) in *Streptomyces* sp. SN-1061M. The presence of highly conserved orthologs within evolutionarily distant species indicates the importance of both regulators and a strong selective pressure. Thus, the regulatory influences of Sco3571 and Sco4385, described in this study, may most likely be transferable to the native producer strains.

In our study, we present the complete set of proteins from the widely used heterologous host strain *S. coelicolor* M512, which bind to the main promoter regions of the caprazamycin and liposidomycin BGC. By either screening the data set for known regulators involved in precursor supply or selecting uncharacterized regulators based on their DACA binding patterns, we identified and characterized Sco3571 and Sco4385 as two new regulators affecting the heterologous production of caprazamycins and liposidomycins. We describe a general approach to identify candidate regulatory genes that need to be deleted or overexpressed to increase production levels of a desired natural product in heterologous systems. In addition, our data strongly imply that regulation of natural product biosynthesis can indeed be cross-linked to the regulation of precursor supply to ensure product formation in a heterologous host.

## MATERIALS AND METHODS

### Bacterial strains, plasmids, oligonucleotides, and culture conditions

All bacterial strains and plasmids are listed in Table S1 in the supplementary material. Standard conditions for the cultivation of *E. coli* were growth in liquid LB medium under agitation at 200 rpm or on LB agar plates, at 37°C. For the cultivation of *Streptomyces* strains, spore stocks of three independent mutants were used to inoculate 50 mL tryptic soy broth (TSB) medium as a pre-culture and grown at 30°C and 200 rpm for 3 days. One milliliter of the pre-culture was used to inoculate 70 mL of production medium (PM) (8), and cultivation was continued under the same conditions for 7 days. All cultivations were carried out in 300 mL baffled Erlenmeyer flasks containing a stainless steel spring. Maintenance of *Streptomyces* was carried out on mannitol soy flour agar plates (35). Apramycin (50 µg/mL), kanamycin (50 µg/mL), tetracycline (12.5 µg/mL), nalidixic acid (25 µg/mL), and carbenicillin (100 µg/mL) were added, if required. If not otherwise stated, all chemicals and media components were purchased from standard commercial sources. Oligonucleotides were purchased from Eurofins Genomics (Ebersberg, Germany) and are listed in Table S2.

### RNA isolation for RNA sequencing

The total RNA was isolated from cultures of three biological replicates of *S. coelicolor* M512/cpzLK09 and *S. coelicolor* M512/lpmLK01, respectively, grown under the aforementioned standard conditions. Samples for RNA isolation were taken after 2 and 4 days of cultivation. Cells were harvested from a culture volume of 5 mL, and RNA was isolated as described below. Following DNase digestion, the RNA was purified using the RNA clean-up kit (Macherey-Nagel, Düren, Germany) and was tested for the absence of DNA by PCR.

### RNA quality control, library preparation, and sequencing

The total RNA was quantified with a Qubit RNA BR Assay Kit (Thermo Fisher), and RNA integrity was checked by an Agilent 2100 BioAnalyzer with the RNA 6000 Pico kit (Agilent). Library preparation was performed with Illumina Stranded Total RNA Prep, Ligation with Ribo-Zero Plus Microbiome according to the manufacturer’s instructions. In brief, 100 ng of total RNA per sample was subjected to rRNA depletion, followed by cDNA library construction, adapter ligation, and 15 cycles of barcoding PCR. Obtained libraries were quantified with Qubit 1x DNA HS Assay Kit (Thermo Fisher), and the fragment distribution was checked by an Agilent 2100 BioAnalyzer using the High Sensitivity DNA Kit (Agilent). Libraries were subsequently pooled and sequenced on an Illumina NovaSeq 6000 device using the NovaSeq 6000 SP Reagent Kit v1.5 (100 cycles) with a run mode of 75, 10, 10, 0. The average number of obtained reads was 27–43 million. The sequencing was demultiplexed with the latest version of the nextflow pipeline: nf-core/demultiplex (36). For demultiplexing, bcl2fastq was used, and the quality was checked with fastp (37).

### RNA-Seq data assessment and analysis

Sequencing statistics including the quality per base and adapter content assessment of the resulting transcriptome sequencing data were conducted with FastQC v0.11.8 (38). All read mappings were performed against the assembled strain *Streptomyces coelicolor* A3(2) (RefSeq ID NC_000964.3) and the respective cosmid sequences (GenBank, accession number GU219978 and FJ490409.1). The mappings of all samples were conducted with HISAT2 v2.1.0 (39). Parameters for spliced alignment of reads were disabled, and strand-specific information was set to reverse complemented (HISAT2 parameter --no-spliced-alignment and --rna-strandness “R”). The resulting mapping files in SAM format were converted to BAM format using SAMtools v1.9 (40). Mapping statistics, including strand specificity estimation and percentage of mapped reads, were conducted with the RNA-Seq module of QualiMap2 v2.2.2-a (41). Gene counts for all samples were computed with featureCounts v1.6.4 (42), where the selected feature type was set to transcript records (featureCounts parameter -t transcript). A quality check for ribosomal rRNA was performed with a self-written script based on the absolute counts of annotated rRNAs. To assess the variability of the replicates of each time series, a principal component analysis (PCA) was conducted with the DESeq2 package v1.28.1 (43). The command “bamCoverage” of DeepTools (44) has been used to generate per-base coverage information for each strand using the aligned reads. The integrated genome browser (IGB) (45) has been used for visualizing the per-base coverage.

### 20 L fermentation of *S. coelicolor* M512/cpzLK09 and *S. coelicolor* M512/lpmLK01

For the acquisition of sufficient protein amounts for the DACA, *S. coelicolor* M512/cpzLK09 and *S. coelicolor* M512/lpmLK01 were grown in 20 L fermenters each. Twenty pre-culture flasks per strain were prepared by inoculation of spores into 50 mL TSB medium. After growth for 2 days, the pre-cultures of each strain were pooled, and 1 L of the pre-culture was used as inoculum for the fermenters (Giovanola b20 fermenter, Giovanola Frères SA, Switzerland) containing 19 L P-medium and 4 mL antifoam, respectively. Temperature was set to 30°C, and aeration rates were 0.5 volumes per fermenter volume per minute (vvm) at 1,000 revolutions per minute. Samples from both fermenters were taken at the following time points: 18, 24, 36, 42, 48, 54, 60, 72, and 90 hours. Depending on the observed cell density at the respective time point, a total sample volume of 550–1,050 mL was taken. A portion (20 mL) of the culture was applied in duplicate for the determination of liponucleoside production rates as described below. One milliliter samples in duplicate were used to determine the cell dry weight (see Extraction and measurement of liponucleosides), and 0.5 mL samples were first stabilized using the RNAprotect bacteria kit (Qiagen, Hilden, Germany) and then stored at −70°C. The residual sampling volume was centrifuged (60 minutes, 4°C, 4,800 × *g*), and resulting cell pellets were washed in 30–50 mL TGED buffer (20 mM Tris-HCl [pH 7.5], 100 mM NaCl, 1 mM EDTA, 0.01% Triton X-100, 10% glycerol, 1 mM DTT). Cells were pelleted again by centrifugation and stored at −70°C until the DACA was conducted.

### RNA isolation, reverse transcription, and RT-qPCR

RNA was isolated from the fermenter samples using the RNeasy plus kit (Qiagen, Hilden, Germany). Cells were transferred into lysing matrix B tubes (2 mL) and lysed in an MP FastPrep-24 (all supplied by MP Biomedicals, USA) for two times, 15 seconds at 4,000 m/s with a cooling step on ice for 4 minutes between the two rounds of shaking. RNA was eluted in RNase-free water and measured at the NanoDrop 1000 spectrophotometer (VWR International, USA). Contaminating DNA was digested using the RNase-free DNase kit (Qiagen, Hilden, Germany). RNA (120 ng) was reverse transcribed into cDNA using the iScript cDNA Synthesis Kit (Bio-Rad Laboratories Inc., USA). Due to small sample volumes, sufficient RNA amounts for reverse transcription were only obtained for samples upward of the 36 hours time point in the case of *S. coelicolor* M512/cpzLK09 and upward of the 48 hours time point in the case of *S. coelicolor* M512/lpmLK01. Subsequently, the qPCR of *cpz10*, *lpmH*, and *hrdB* (for primers, see Table S2) was conducted in technical duplicate on a Bio-Rad MyiQ2 thermocycler. The PCR reaction volume of 20 µL was composed of 10 µL 2x QuantiNova SYBR Green PCR master mix (Qiagen, Hilden, Germany), 0.7 µM forward and reverse primer each, water, and either 12 ng cDNA, gDNA (positive control), or test RNA (negative control) as templates. The cycler protocol according to the QuantiNova SYBR Green PCR handbook was the following: initial activation step for 2 minutes at 95°C followed by 40 cycles of a 5 second denaturation step at 95°C and 10 seconds of combined annealing/extension step at 60°C. A subsequent melting curve analysis over a temperature range of 55–95°C in 0.5°C steps was performed to validate the specificity of the resulting PCR product. C(T) values for each sample were supplied by the MyiQ2 real-time PCR detection software (Bio-Rad Laboratories Inc., USA). Relative transcription rates of *cpz10* and *lpmH* were normalized against the expression rates of the reference gene *hrdB* using the cycle threshold method (46).

### DNA-affinity-capturing assay (DACA)

Extended intergenic regions, flanked by fragments of the down- and upstream genes, were amplified by PCR. Amplicons from the caprazamycin gene cluster were designated as P*cpz6* (424 bp), P*cpz9* (358 bp), P*cpz10* (365 bp), P*cpz24* (431 bp), and those of the liposidomycin gene cluster as P*lpmD* (544 bp), P*lpmG* (520 bp)*,* P*lpmH* (369 bp), and P*lpmV* (455 bp). The promoter region of the vegetative σ-factor HrdB, assigned as P*hrdB* (559 bp), served as a negative control. The sequences of the DNA fragments were confirmed by sequencing. Biotinylation in a second PCR was conducted as described previously (15). Purified DNA (120 µg) of each promoter was used in the DACA. Culture samples from time points 36 and 54 hours for *S. coelicolor* M512/cpzLK09 and time points 48 and 72 hours for *S. coelicolor* M512/lpmLK01 were used to extract proteins. Cell disruption was achieved by passaging the cells through the French press four times at 1,000 psi. Individual steps of the DACA experiment were conducted as described previously (15).

### Processing of the DACA protein samples and identification of proteins via NanoLC-MS/MS analysis

Proteins were purified on a NuPAGE 12% gel (Invitrogen), and Coomassie-stained gel pieces were digested in gel with trypsin (47). Desalted peptide mixtures (48) were separated on an Easy-nLC 1200 system coupled to a quadrupole Orbitrap Exploris 480 mass spectrometer (all Thermo Fisher Scientific) as described previously (49) with slight modifications: peptides were separated using a 57 minute segmented gradient from 10%–90% of solvent B (80% acetonitrile in 0.1% formic acid) in solvent A (0.1% formic acid) at a flow rate of 200 nL/minute. The mass spectrometer was operated in a data‐dependent mode, collecting MS spectra in the Orbitrap mass analyzer (60,000 resolution, 300–1,750 *m/z* range) with an automatic gain control (AGC) set to standard and a maximum ion injection time set to automatic. The 12 most intense precursor ions were sequentially fragmented with a normalized collision energy of 28 in each scan cycle using higher energy collisional dissociation (HCD) fragmentation. In all measurements, sequenced precursor masses were excluded from further selection for 30 seconds. MS/MS spectra were recorded with a resolution of 30,000, whereby fill time was set to automatic.

Acquired MS spectra were processed with the MaxQuant software package version 1.6.14.0 (50) with integrated Andromeda search engine (51). A database search was performed against a *Streptomyces coelicolor* database obtained from Uniprot (https://www.uniprot.org/, last access: 10 July 2022, 8,039 protein entries), sequences of the liposidomycin and caprazamycin cluster proteins, and 286 commonly observed contaminants. Endoprotease trypsin was defined as a protease with a maximum of two missed cleavages. Oxidation of methionine and protein N-terminal acetylation were specified as variable modifications. Carbamidomethylation on cysteine was set as fixed modification. Mass tolerance was set to 4.5 parts per million (ppm) for precursor ions and 20 ppm for fragment ions. Peptide, protein, and modification site identifications were reported at a false discovery rate (FDR) of 0.01, estimated by the target-decoy approach (52). The Intensity-Based Absolute Quantification (iBAQ) and Label-Free Quantification (LFQ) algorithms were enabled (53).

### Construction of gene overexpression strains

Genes *sco2987*, *sco3571*, *sco4385*, *sco5956, cpz9*, and *lpmG* were amplified from the genomic DNA of *S. coelicolor* M512/cpzLK09 and *S. coelicolor* M512/lpmLK01, respectively, thereby attaching *Hind*III and *Spe*I restriction sites. The genes were individually cloned into the replicating high-copy vector pUWL-apra-oriT under the control of the strong constitutive promoter ermE*. All generated constructs pSW4 (*sco2987*), pSW5 (*sco3571*), pSW6 (*sco4385*), pSW7 (*sco5956*), pSW8 (*cpz9*), and pSW9 (*lpmG*) were verified by sequencing and transferred into *S. coelicolor* M512 harboring either the caprazamycin or liposidomycin gene cluster, taking advantage of the non-DNA-methylating *E. coli* strain ET12567/pUZ8002 as shuttle. Exconjugants were tested for the presence of the plasmid by colony PCR using the primer pair pUWL_test_fwd/pUWL_test_rev. Production levels of three independent clones were assessed by extraction of the spent medium and measurement by LC-MS as described below.

### Gene deletions via plasmid-based CRISPR-Cas9 approach

For the deletion of genes *sco3571* and *sco4385*, a plasmid-based CRISPR-Cas9 approach was applied. A suitable spacer sequence of 20 bp within the target genes was identified using the online tool CRISPY-web (https://crispy.secondarymetabolites.org) (54). This sequence was incorporated into the forward oligonucleotide for amplification of the whole sgRNA from the pCRISPR-TT vector, and subsequently, the PCR product was cloned back into pCRISPR-TT (via *Sna*BI and *Nco*I restriction sites) thereby inserting the 20 bp spacer sequence. Using genomic DNA, approximately 1,000 bp of the up- and downstream region of the target genes were amplified, which would function as homologous arms to repair the Cas9-induced DNA cleavage. Primers were designed for Gibson assembly to fuse the up- and downstream homology domains into the *Eco*RV restriction site of pBlueScript SK II (+), which allows blue/white screening of the transformants. Clones containing the successfully assembled domains were identified using the primer pair pSET152_test_fwd/pSET152_test_rev and were checked by sequencing. The assembled homology domains were excised from pBlueScript SK II (+) and purified by gel extraction (QIAquick Gel Extraction Kit, Qiagen, Hilden, Germany). Blunt end ligation was carried out into the *Stu*I restriction site of the pCRISPR-TT-spacer constructs. Successful cloning was verified by colony PCR using the primer pair pCRISPR-TT_test_Stu_fwd/pCRISPR-TT_test_Stu_rev and sequencing of the plasmids. Validated constructs were assigned pSW18 and pSW20 for the deletion of *sco4385* and *sco3571*, respectively. Plasmids were introduced into *S. coelicolor* M512/cpzLK09 and *S. coelicolor* M512/lpmLK01 by biparental conjugation, and spores were obtained for further analysis. Induction of the *cas9* gene under the control of a theophylline-inducible riboswitch was achieved by plating the spores on TSB-agar plates containing 10 mM theophylline and apramycin. Single colonies were tested for the deletion of the target genes by colony PCR, and positive mutants were confluently streaked to obtain spore suspensions. After several attempts and repeated *cas9* inductions, only two colonies were tested positive for deletion of *sco3571* in both target strains, respectively. Finally, the plasmid was cured from the cells in one to two rounds by incubating the streaked TSB-agar plates at 37°C, which prevents pSW18 and pSW20 from replicating. Mutant strains were tested for the loss of the vector by streaking them on plates with and without apramycin. In the case of one exconjugant of *S. coelicolor*/lpmLK01/Δ*sco4385*, *S. coelicolor*/lpmLK01/Δ*sco3571*, and *S. coelicolor*/cpzLK09/Δ*sco3571*, respectively, apramycin resistance was still given after five rounds of curing. These mutants were used for production determination still carrying the plasmid, while all other mutant strains had successfully lost the plasmid. Where possible, three independent clones were used to determine the production levels of the deletion strains.

### Extraction and LC-MS measurement of liponucleosides

To assess the levels of liponucleoside production, the overexpression and gene deletion mutants of *S. coelicolor* M512/cpzLK09 and *S. coelicolor* M512/lpmLK01 were cultivated under standard conditions as described above. After 7 days of growth of the main culture, cell dry weight was determined in duplicate by centrifuging 1 mL of the culture in 1.5 mL microcentrifuge tubes (pre-incubated at 80°C), washing the cell pellet with ddH_2_O, centrifuging it again, and then drying the resulting cell pellet for at least 48 hours at 80°C. Additionally, 50 mL of the culture was harvested by centrifugation (15 minutes, room temperature [RT], 4,000 × *g*), 40 mL of the supernatant was acidified to pH 4 using HCl, and then extracted with the same volume of *n*-butanol. After evaporation of the organic phase, remaining residues were dissolved in 1 mL of LC-MS grade methanol and were measured by HPLC-ESI-MS, as described previously (20). Liponucleoside levels were correlated to the cell dry weight.

### Gene overexpression, protein purification, and size exclusion chromatography of Sco4385

Gene *sco4385* was amplified using the primer pair sco4385_EcoRI_fwd/sco4385_HindIII_rev and was cloned into the corresponding restriction sites of the pHis8 vector. Successful cloning yielding plasmid pSW21 was confirmed by sequencing (Eurofins Genomics, Ebersberg, Germany). Chemically competent *E. coli* BL21 DE3 was transformed with pSW21 and used to inoculate 4 mL LB medium (containing kanamycin). For protein overproduction of His-tagged Sco4385, this culture was used to inoculate 100 mL of terrific broth medium to an OD_600_ of 0.05. Incubation was continued under standard conditions as mentioned before. The culture was induced at an OD_600_ of 0.7 with 0.5 mM IPTG. Growth was continued at 20°C for 16 hours. Cells were harvested, washed in TGED buffer (see above), and pellets were stored at −20°C. For protein isolation, cells were resuspended in lysis buffer (50 mM Tris-HCl [pH 7.5], 500 mM NaCl, 10% glycerol, 10 mM β-mercaptoethanol, 1% TWEEN-20) and disrupted by French press. Centrifugation (1 hour, 4°C, 15,000 × *g*) removed cell debris, and the clear lysate was loaded onto a Ni-NTA hand column (500 µL CV). Five volumes of running buffer (50 mM Tris-HCl [pH 7.5], 500 mM NaCl, 10% glycerol, 1 mM DTT, and 20 mM) were added sequentially to the column for washing. And an imidazole gradient (running buffer containing 100, 150, 200, or 250 mM imidazole) was applied stepwise (1 CV each) to elute Sco4385 protein from the column. All fractions containing solely the desired protein and no contaminating bands on the SDS gel were pooled and applied to size exclusion chromatography (running buffer: 50 mM Tris-HCl [pH 7.5], 250 mM NaCl; flow rate: 0.75 mL/minute; temperature: 4°C). All 1 mL fractions containing Sco4385 were pooled, concentrated in Amicon (Merck, Darmstadt), and glycerol was added to a final concentration of 10% to allow storage at −70°C.

### Size exclusion chromatography with multi-angle light scattering (SEC-MALS) of Sco4385

To further characterize Sco4385 with regard to its multimeric state, analytical SEC-MALS measurements were performed. For this purpose, the protein was purified, as previously described, and applied to an Äkta micro (GE Healthcare) coupled with a MiniDAWN Treos system (Wyatt Technology). Size exclusion chromatography was conducted at room temperature on a Superose 6 increase 10/300 GL size exclusion column with a running buffer consisting of 50 mM Tris-HCl (pH 7.5), 250 mM NaCl, and at a flow rate of 0.4 mL/minute. The concentration signal was determined by an Optilab T-rex refractometer (Wyatt Technology). A separate SPD-20A Prominence UV/VIS detector (Shimadzu Corporation) was utilized for detection at 280 nm. The experimental data were analyzed with the software ASTRA 7 (Wyatt Technology).

### Biolayer interferometry (BLI)

DNA oligomers (50 bp) covering the whole P*cpz10* and P*lpmH* promoter region tested for Sco4385 binding were purchased from Eurofins Genomics (Ebersberg, Germany). The sequences of all oligonucleotides are listed in Table S3. Generation of double-stranded DNA of these oligomers carrying a single-stranded complementary overhang to the ReDCaT linker sequence (20 nt; for sequence, see Table S3) was achieved following a previously described protocol (22). Biolayer-interferometry measurements were conducted on an Octet K2 system (Pall FortéBio LLC, Fremont, CA, USA) using streptavidin-covered “dip-and-read” biosensors (SA sensors, same supplier) at 30°C and a shaking speed of 1,000 rpm. Sensors were first equilibrated in binding buffer (50 mM Tris-HCl [pH 7.5], 150 mM NaCl) for 5 minutes prior to the measurement. The samples were prepared in a black 96-well plate for sequential dipping of the sensor into the wells, each containing 230 µL sample or buffer volume. Both biotinylated linker and pre-annealed dsDNA were applied at a concentration of 1 µM. For baseline measurement, the sensor was dipped into binding buffer for 60 seconds while all loading or protein binding steps were each held for 180 seconds. Purified Sco4385 protein was diluted to a final concentration of 1 µM using the binding buffer. All measurements for determining the binding site were conducted in technical duplicate (Tables S4 and S5). The experiment was repeated under the same conditions with oligonucleotides containing the predicted Sco4385 binding sites (Table S7).

### Computational generation of the consensus sequence and search for alternative binding sites

A common consensus sequence, based on the segment sequences bound by Sco4385, was identified by GLAM2 (24), which is an integrated tool of the MEME Suite (55). Using the generated consensus sequence and the RSAT tool “dna-pattern” (56), the genome of *S. coelicolor* M512 was screened for alternative binding sites of Sco4385.

## Data Availability

The data discussed in this publication have been deposited in NCBI’s Gene Expression Omnibus (57, 58) and are accessible through the GEO Series accession number GSE291227.

## SUPPLEMENTAL MATERIAL

Data Set S1 aem.02315-24-s0001.xlsx DACA list of detected proteins.

Supplemental figures and tables aem.02315-24-s0002.pdf Figures S1 to S12 and Tables S1 to S7.

ASM does not own the copyrights to Supplemental Material that may be linked to, or accessed through, an article. The authors have granted ASM a non-exclusive, world-wide license to publish the Supplemental Material files. Please contact the corresponding author directly for reuse.

## References

[1] Igarashi M, Nakagawa N, Doi N, Hattori S, Naganawa H, Hamada M. 2003. Caprazamycin B, a Novel Anti-tuberculosis Antibiotic, from Streptomyces sp. J Antibiot 56:580–583. doi:10.7164/antibiotics.56.580 12931868

[2] Takeuchi T, Igarashi M, Naganawa H, Hamada M. 2004. Antibiotic caprazamycins and process for producing the same. Patent no. 6,780,616. USA

[3] Takahashi Y, Igarashi M, Miyake T, Soutome H, Ishikawa K, Komatsuki Y, Koyama Y, Nakagawa N, Hattori S, Inoue K, Doi N, Akamatsu Y. 2013. Novel semisynthetic antibiotics from caprazamycins A–G: caprazene derivatives and their antibacterial activity. J Antibiot 66:171–178. doi:10.1038/ja.2013.9 23532021

[4] Stewart IE, Durham PG, Sittenauer JM, Barreda AP, Stowell GW, Moody C, Mecham JB, Simpson C, Daily S, Maloney SE, Williams MD, Severynse-Stevens D, Hickey AJ. 2022. Optimization and scale up of spray dried CPZEN-45 aerosol powders for inhaled tuberculosis treatment. Pharm Res 39:3359–3370. doi:10.1007/s11095-022-03393-w 36114362 PMC9483285

[5] Kimura K, Miyata N, Kawanishi G, Kamio Y, Izaki K, Isono K. 1989. Liposidomycin C inhibits phospho-N-acetylmuramyl-pentapeptide transferase in peptidoglycan synthesis of Escherichia coli Y-10. Agric Biol Chem 53:1811–1815. doi:10.1271/bbb1961.53.1811

[6] Mashalidis EH, Kaeser B, Terasawa Y, Katsuyama A, Kwon DY, Lee K, Hong J, Ichikawa S, Lee SY. 2019. Chemical logic of MraY inhibition by antibacterial nucleoside natural products. Nat Commun 10:2917. doi:10.1038/s41467-019-10957-9 31266949 PMC6606608

[7] Kaysser L, Siebenberg S, Kammerer B, Gust B. 2010. Analysis of the liposidomycin gene cluster leads to the identification of new caprazamycin derivatives. Chembiochem 11:191–196. doi:10.1002/cbic.200900637 20039253

[8] Kaysser L, Lutsch L, Siebenberg S, Wemakor E, Kammerer B, Gust B. 2009. Identification and manipulation of the caprazamycin gene cluster lead to new simplified liponucleoside antibiotics and give insights into the biosynthetic pathway. J Biol Chem 284:14987–14996. doi:10.1074/jbc.M901258200 19351877 PMC2685681

[9] Tang X, Eitel K, Kaysser L, Kulik A, Grond S, Gust B. 2013. A two-step sulfation in antibiotic biosynthesis requires A type III polyketide synthase. Nat Chem Biol 9:610–615. doi:10.1038/nchembio.1310 23912167

[10] Kaysser L, Wemakor E, Siebenberg S, Salas JA, Sohng JK, Kammerer B, Gust B. 2010. Formation and attachment of the deoxysugar moiety and assembly of the gene cluster for caprazamycin biosynthesis. Appl Environ Microbiol 76:4008–4018. doi:10.1128/AEM.02740-09 20418426 PMC2893494

[11] Chi X, Pahari P, Nonaka K, Van Lanen SG. 2011. Biosynthetic origin and mechanism of formation of the aminoribosyl moiety of peptidyl nucleoside antibiotics. J Am Chem Soc 133:14452–14459. doi:10.1021/ja206304k 21819104 PMC3174061

[12] Barnard-Britson S, Chi X, Nonaka K, Spork AP, Tibrewal N, Goswami A, Pahari P, Ducho C, Rohr J, Van Lanen SG. 2012. Amalgamation of nucleosides and amino acids in antibiotic biosynthesis: discovery of an L-threonine:uridine-5’-aldehyde transaldolase. J Am Chem Soc 134:18514–18517. doi:10.1021/ja308185q 23110675 PMC3528340

[13] Wiker F, Hauck N, Grond S, Gust B. 2019. Caprazamycins: biosynthesis and structure activity relationship studies. Int J Med Microbiol 309:319–324. doi:10.1016/j.ijmm.2019.05.004 31138496

[14] McErlean M, Liu X, Cui Z, Gust B, Van Lanen SG. 2021. Identification and characterization of enzymes involved in the biosynthesis of pyrimidine nucleoside antibiotics. Nat Prod Rep 38:1362–1407. doi:10.1039/d0np00064g 33404015 PMC9483940

[15] Bekiesch P, Franz-Wachtel M, Kulik A, Brocker M, Forchhammer K, Gust B, Apel AK. 2016. DNA affinity capturing identifies new regulators of the heterologously expressed novobiocin gene cluster in Streptomyces coelicolor M512. Appl Microbiol Biotechnol 100:4495–4509. doi:10.1007/s00253-016-7306-1 26795961

[16] Bekiesch P, Forchhammer K, Apel AK. 2016. Characterization of DNA binding sites of RokB, a ROK-family regulator from streptomyces coelicolor reveals the RokB regulon. PLoS ONE 11:e0153249. doi:10.1371/journal.pone.0153249 27145180 PMC4856308

[17] Park SS, Yang YH, Song E, Kim EJ, Kim WS, Sohng JK, Lee HC, Liou KK, Kim BG. 2009. Mass spectrometric screening of transcriptional regulators involved in antibiotic biosynthesis in Streptomyces coelicolor A3(2). J Ind Microbiol Biotechnol 36:1073–1083. doi:10.1007/s10295-009-0591-2 19468766

[18] Martin RG, Rosner JL. 2001. The AraC transcriptional activators. Curr Opin Microbiol 4:132–137. doi:10.1016/s1369-5274(00)00178-8 11282467

[19] Augustijn HE, Karapliafis D, Joosten KMM, Rigali S, van Wezel GP, Medema MH. 2024. LogoMotif: a comprehensive database of transcription factor binding site profiles in Actinobacteria. J Mol Biol 436:168558. doi:10.1016/j.jmb.2024.168558 38580076

[20] Bär D, Konetschny B, Kulik A, Xu H, Paccagnella D, Beller P, Ziemert N, Dickschat JS, Gust B. 2022. Origin of the 3-methylglutaryl moiety in caprazamycin biosynthesis. Microb Cell Fact 21:232. doi:10.1186/s12934-022-01955-6 36335365 PMC9636800

[21] Gao C, Mulder D, Yin C, Elliot MA. 2012. Crp is a global regulator of antibiotic production in streptomyces. MBio 3:e00407–e00412. doi:10.1128/mBio.00407-12 23232715 PMC3520106

[22] Stevenson CEM, Assaad A, Chandra G, Le TBK, Greive SJ, Bibb MJ, Lawson DM. 2013. Investigation of DNA sequence recognition by a streptomycete MarR family transcriptional regulator through surface plasmon resonance and X-ray crystallography. Nucleic Acids Res 41:7009–7022. doi:10.1093/nar/gkt523 23748564 PMC3737563

[23] Orthwein T, Huergo LF, Forchhammer K, Selim KA. 2021. Kinetic analysis of a protein-protein complex to determine its dissociation constant (K_D_) and the effective concentration (EC_50_) of an interplaying effector molecule using bio-layer interferometry. Bio Protoc 11:e4152. doi:10.21769/BioProtoc.4152 PMC8443453 34604457

[24] Frith MC, Saunders NFW, Kobe B, Bailey TL. 2008. Discovering sequence motifs with arbitrary insertions and deletions. PLoS Comput Biol 4:e1000071. doi:10.1371/journal.pcbi.1000071 18437229 PMC2323616

[25] Myronovskyi M, Rosenkränzer B, Nadmid S, Pujic P, Normand P, Luzhetskyy A. 2018. Generation of a cluster-free Streptomyces albus chassis strains for improved heterologous expression of secondary metabolite clusters. Metab Eng 49:316–324. doi:10.1016/j.ymben.2018.09.004 30196100

[26] Schwarz PN, Buchmann A, Roller L, Kulik A, Gross H, Wohlleben W, Stegmann E. 2018. The immunosuppressant brasilicardin: determination of the biosynthetic gene cluster in the heterologous host Amycolatopsis japonicum. Biotechnol J 13:1–38. doi:10.1002/biot.201700527 29045029

[27] Beller P, Fink P, Wolf F, Männle D, Helmle I, Kuttenlochner W, Unterfrauner D, Engelbrecht A, Staudt ND, Kulik A, Groll M, Gross H, Kaysser L. 2024. Characterization of the cystargolide biosynthetic gene cluster and functional analysis of the methyltransferase CysG. J Biol Chem 300:105507. doi:10.1016/j.jbc.2023.105507 38029966 PMC10776993

[28] Ahn SK, Cuthbertson L, Nodwell JR. 2012. Genome context as a predictive tool for identifying regulatory targets of the TetR family transcriptional regulators. PLoS One 7:e50562. doi:10.1371/journal.pone.0050562 23226315 PMC3511530

[29] Murarka P, Bagga T, Singh P, Rangra S, Srivastava P. 2019. Isolation and identification of a TetR family protein that regulates the biodesulfurization operon. AMB Express 9:71. doi:10.1186/s13568-019-0801-x 31127394 PMC6534649

[30] Wei K, Wu Y, Li L, Jiang W, Hu J, Lu Y, Chen S. 2018. MilR2, a novel TetR family regulator involved in 5-oxomilbemycin A3/A4 biosynthesis in Streptomyces hygroscopicus. Appl Microbiol Biotechnol 102:8841–8853. doi:10.1007/s00253-018-9280-2 30121749

[31] Yuan PH, Zhou RC, Chen X, Luo S, Wang F, Mao XM, Li YQ. 2016. DepR1, a TetR family transcriptional regulator, positively regulates daptomycin production in an industrial producer, Streptomyces roseosporus SW0702. Appl Environ Microbiol 82:1898–1905. doi:10.1128/AEM.03002-15 26773081 PMC4784024

[32] Jiang MX, Yin M, Wu SH, Han XL, Ji KY, Wen ML, Lu T. 2017. GdmRIII, a TetR family transcriptional regulator, controls geldanamycin and elaiophylin biosynthesis in Streptomyces autolyticus CGMCC0516. Sci Rep 7:4803. doi:10.1038/s41598-017-05073-x 28684749 PMC5500506

[33] Cuthbertson L, Nodwell JR. 2013. The TetR family of regulators. Microbiol Mol Biol Rev 77:440–475. doi:10.1128/MMBR.00018-13 24006471 PMC3811609

[34] Mirdita M, Schütze K, Moriwaki Y, Heo L, Ovchinnikov S, Steinegger M. 2022. ColabFold: making protein folding accessible to all. Nat Methods 19:679–682. doi:10.1038/s41592-022-01488-1 35637307 PMC9184281

[35] Kieser T, Bibb MJ, Buttner MJ, Chater KF, Hopwood DA. 2000. Practical Streptomyces genetics. John Innes Foundation Norwich.

[36] Ewels PA, Peltzer A, Fillinger S, Patel H, Alneberg J, Wilm A, Garcia MU, Di Tommaso P, Nahnsen S. 2020. The nf-core framework for community-curated bioinformatics pipelines. Nat Biotechnol 38:276–278. doi:10.1038/s41587-020-0439-x 32055031

[37] Chen S, Zhou Y, Chen Y, Gu J. 2018. Fastp: an ultra-fast all-in-one FASTQ preprocessor. J Bioinform 34:i884–i890. doi:10.1093/bioinformatics/bty560 PMC6129281 30423086

[38] Andrews S. 2024. FastQC: a quality control tool for high throughput sequence data. Available from: http://www.bioinformatics.babraham.ac.uk/projects/fastqc. Retrieved 22 May 2024.

[39] Kim D, Langmead B, Salzberg SL. 2015. HISAT: a fast spliced aligner with low memory requirements. Nat Methods 12:357–360. doi:10.1038/nmeth.3317 25751142 PMC4655817

[40] Li H, Handsaker B, Wysoker A, Fennell T, Ruan J, Homer N, Marth G, Abecasis G, Durbin R, 1000 Genome Project Data Processing Subgroup. 2009. The sequence Alignment/Map format and SAMtools. J Bioinform 25:2078–2079. doi:10.1093/bioinformatics/btp352 PMC2723002 19505943

[41] Okonechnikov K, Conesa A, García-Alcalde F. 2016. Qualimap 2: advanced multi-sample quality control for high-throughput sequencing data. J Bioinform 32:292–294. doi:10.1093/bioinformatics/btv566 PMC4708105 26428292

[42] Liao Y, Smyth GK, Shi W. 2014. featureCounts: an efficient general purpose program for assigning sequence reads to genomic features. J Bioinform 30:923–930. doi:10.1093/bioinformatics/btt656 24227677

[43] Love MI, Huber W, Anders S. 2014. Moderated estimation of fold change and dispersion for RNA-seq data with DESeq2. Genome Biol 15:550. doi:10.1186/s13059-014-0550-8 25516281 PMC4302049

[44] Quinlan AR, Hall IM. 2010. BEDTools: a flexible suite of utilities for comparing genomic features. J Bioinform 26:841–842. doi:10.1093/bioinformatics/btq033 PMC2832824 20110278

[45] Freese NH, Norris DC, Loraine AE. 2016. Integrated genome browser: visual analytics platform for genomics. J Bioinform 32:2089–2095. doi:10.1093/bioinformatics/btw069 PMC4937187 27153568

[46] Livak KJ, Schmittgen TD. 2001. Analysis of relative gene expression data using real-time quantitative PCR and the 2(-Delta Delta C(T)) method. Methods 25:402–408. doi:10.1006/meth.2001.1262 11846609

[47] Borchert N, Dieterich C, Krug K, Schütz W, Jung S, Nordheim A, Sommer RJ, Macek B. 2010. Proteogenomics of Pristionchus pacificus reveals distinct proteome structure of nematode models. Genome Res 20:837–846. doi:10.1101/gr.103119.109 20237107 PMC2877580

[48] Rappsilber J, Mann M, Ishihama Y. 2007. Protocol for micro-purification, enrichment, pre-fractionation and storage of peptides for proteomics using stagetips. Nat Protoc 2:1896–1906. doi:10.1038/nprot.2007.261 17703201

[49] Bekker-Jensen DB, Martínez-Val A, Steigerwald S, Rüther P, Fort KL, Arrey TN, Harder A, Makarov A, Olsen JV. 2020. A compact quadrupole-orbitrap mass spectrometer with FAIMS Interface improves proteome coverage in short LC gradients. Mol Cell Proteomics 19:716–729. doi:10.1074/mcp.TIR119.001906 32051234 PMC7124470

[50] Cox J, Mann M. 2008. MaxQuant enables high peptide identification rates, individualized p.p.b.-range mass accuracies and proteome-wide protein quantification. Nat Biotechnol 26:1367–1372. doi:10.1038/nbt.1511 19029910

[51] Cox J, Neuhauser N, Michalski A, Scheltema RA, Olsen JV, Mann M. 2011. Andromeda: a peptide search engine integrated into the MaxQuant environment. J Proteome Res 10:1794–1805. doi:10.1021/pr101065j 21254760

[52] Elias JE, Gygi SP. 2007. Target-decoy search strategy for increased confidence in large-scale protein identifications by mass spectrometry. Nat Methods 4:207–214. doi:10.1038/nmeth1019 17327847

[53] Tyanova S, Temu T, Cox J. 2016. The MaxQuant computational platform for mass spectrometry-based shotgun proteomics. Nat Protoc 11:2301–2319. doi:10.1038/nprot.2016.136 27809316

[54] Blin K, Pedersen LE, Weber T, Lee SY. 2016. CRISPy-web: an online resource to design sgRNAs for CRISPR applications. Synth Syst Biotechnol 1:118–121. doi:10.1016/j.synbio.2016.01.003 29062934 PMC5640694

[55] Bailey TL, Johnson J, Grant CE, Noble WS. 2015. The MEME suite. Nucleic Acids Res 43:W39–49. doi:10.1093/nar/gkv416 25953851 PMC4489269

[56] Santana-Garcia W, Castro-Mondragon JA, Padilla-Gálvez M, Nguyen NTT, Elizondo-Salas A, Ksouri N, Gerbes F, Thieffry D, Vincens P, Contreras-Moreira B, van Helden J, Thomas-Chollier M, Medina-Rivera A. 2022. RSAT 2022: regulatory sequence analysis tools. Nucleic Acids Res 50:W670–W676. doi:10.1093/nar/gkac312 35544234 PMC9252783

[57] Edgar R, Domrachev M, Lash AE. 2002. Gene expression omnibus: NCBI gene expression and hybridization array data repository. Nucleic Acids Res 30:207–210. doi:10.1093/nar/30.1.207 11752295 PMC99122

[58] Barrett T, Wilhite SE, Ledoux P, Evangelista C, Kim IF, Tomashevsky M, Marshall KA, Phillippy KH, Sherman PM, Holko M, Yefanov A, Lee H, Zhang N, Robertson CL, Serova N, Davis S, Soboleva A. 2013. NCBI GEO: archive for functional genomics data sets--update. Nucleic Acids Res 41:D991–5. doi:10.1093/nar/gks1193 23193258 PMC3531084

