# Supplementary material for: The TetR-like regulator Sco4385 and Crp-like regulator Sco3571 modulate heterologous production of antibiotics in Streptomyces coelicolor M512
Source: Appl Environ Microbiol. 2025 Apr 4;91(5):e02315-24. doi: 10.1128/aem.02315-24 (PMC12093943; doi:10.1128/aem.02315-24)
Supplement: Supplemental figures and tables — Figures S1 to S12 and Tables S1 to S7. [file aem.02315-24-s0002.pdf]

Supporting information

The TetR-like regulator Sco4385 and Crp-like regulator Sco3571 modulate heterologous production of antibiotics in *Streptomyces coelicolor* M512

Sarah Wilcken,[1,2] Panagiota-Hanna Koutsandrea,[1] Tomke Bakker,[1] Andreas Kulik,[3] Tim Orthwein,[4] Mirita Franz-Wachtel,[5] Theresa Harbig,[6] Kay Katja Nieselt,[6] Karl Forchhammer,[4] Heike Brötz-Oesterhelt,[2,3,7] Boris Macek,[5] Silja Mordhorst,[1] Leonard Kaysser,[8,#] Bertolt Gust[1,2,#]

[1] Pharmaceutical Biology, Pharmaceutical Institute, Eberhard-Karls-University Tübingen, Auf der Morgenstelle 8, 72076 Tübingen, Germany

[2] German Centre for Infection Research (DZIF), Partner site Tübingen, Tübingen, Germany

[3] Department of Microbial Bioactive Compounds, Interfaculty Institute of Microbiology and Infection Medicine, Eberhard-Karls University Tübingen, Auf der Morgenstelle 28, 72076 Tubingen, Germany

[4] Department of Microbiology and Organismic Interactions, Interfaculty Institute of Microbiology and Infection Medicine, Eberhard-Karls-University Tübingen, Auf der Morgenstelle 28, 72076 Tübingen, Germany

[5] Proteome Center Tübingen, Institute of Cell Biology, Eberhard-Karls-University Tübingen, Auf der Morgenstelle 15, 72076 Tübingen, Germany

[6] Interfaculty Institute for Bioinformatics and Medical Informatics, Eberhard-Karls-University Tübingen, Sand 14, 72076 Tübingen, Germany

[7] Cluster of Excellence Controlling Microbes to Fight Infections, University of Tübingen, Auf der Morgenstelle 28, 72076 Tübingen, Germany

[8] Institute for Drug Discovery, Department of Pharmaceutical Biology, Leipzig University, Eilenburger Str. 14, 04317 Leipzig, Germany

[#]Address correspondence to Bertolt Gust, bertolt.gust@uni-tuebingen.de.

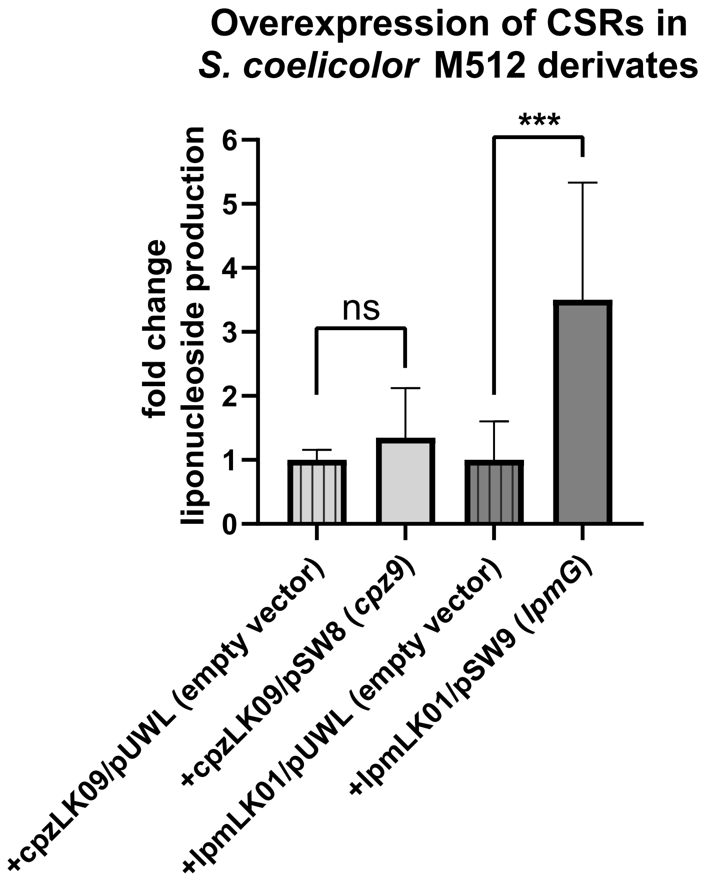

Figure S1: Liponucleoside production upon overexpression of the cluster-situated regulators (CSRs) Cpz9 and LpmG in *S. coelicolor* M512 harboring the caprazamycin (cpzLK09) or liposidomycin (lpmLK01) biosynthetic gene cluster, respectively. Bars indicate mean production levels and error bars indicate the production range of three independent biological replicates. Statistically significant differences were tested using unpaired two-tailed t-tests, where *** signifies p-value <0.001. ns: not significant.

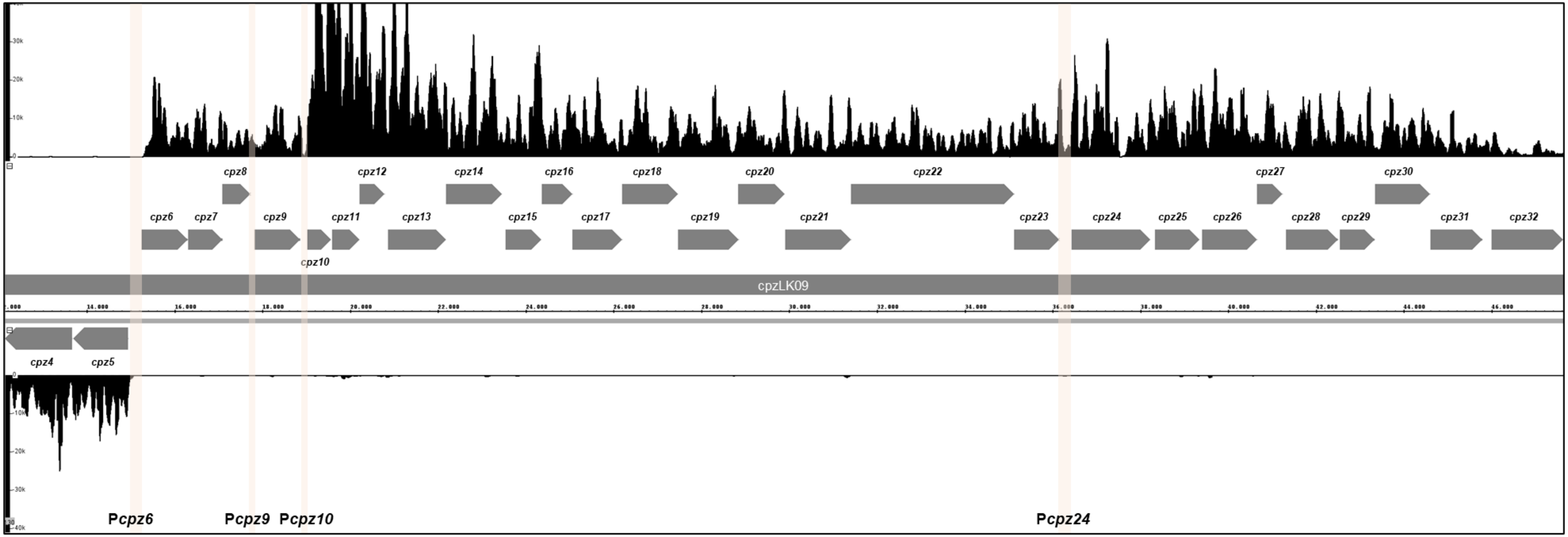

Figure S2: Transcription of the caprazamycin gene cluster in *S. coelicolor* M512/cpzLK09 after two days of cultivation. The genes of the cluster, depicted as arrows, are divided into sense and anti-sense strand and respective coverage plots are mapped against them. A threshold value of 40000 read counts is applied. Intergenic regions selected for DNA-affinity-capturing assays are labelled (P*cpz6*, P*cpz9*, P*cpz10* and P*cpz24*) and highlighted with light red bars.

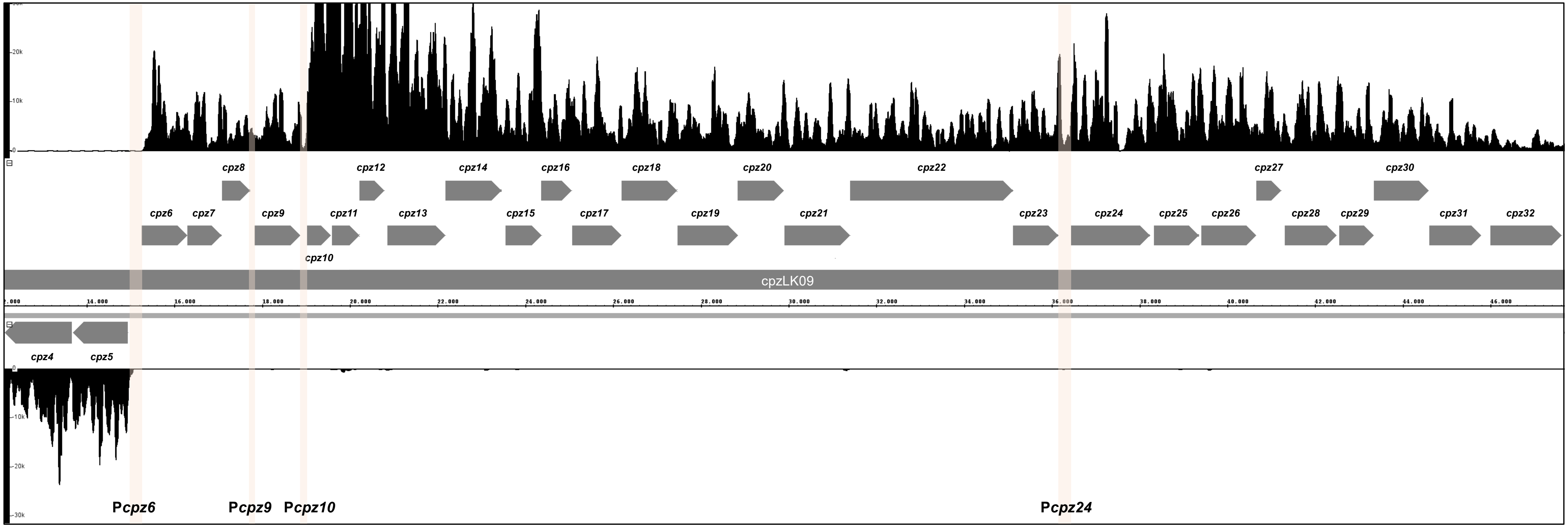

Figure S3: Transcription of the caprazamycin gene cluster in *S. coelicolor* M512/cpzLK09 after four days of cultivation. The genes of the cluster, depicted as arrows, are divided into sense and anti-sense strand and respective coverage plots are mapped against them. A threshold value of 30000 read counts is applied. Intergenic regions selected for DNA-affinity-capturing assays are labelled (P*cpz6*, P*cpz9*, P*cpz10* and P*cpz24*) and highlighted with light red bars.

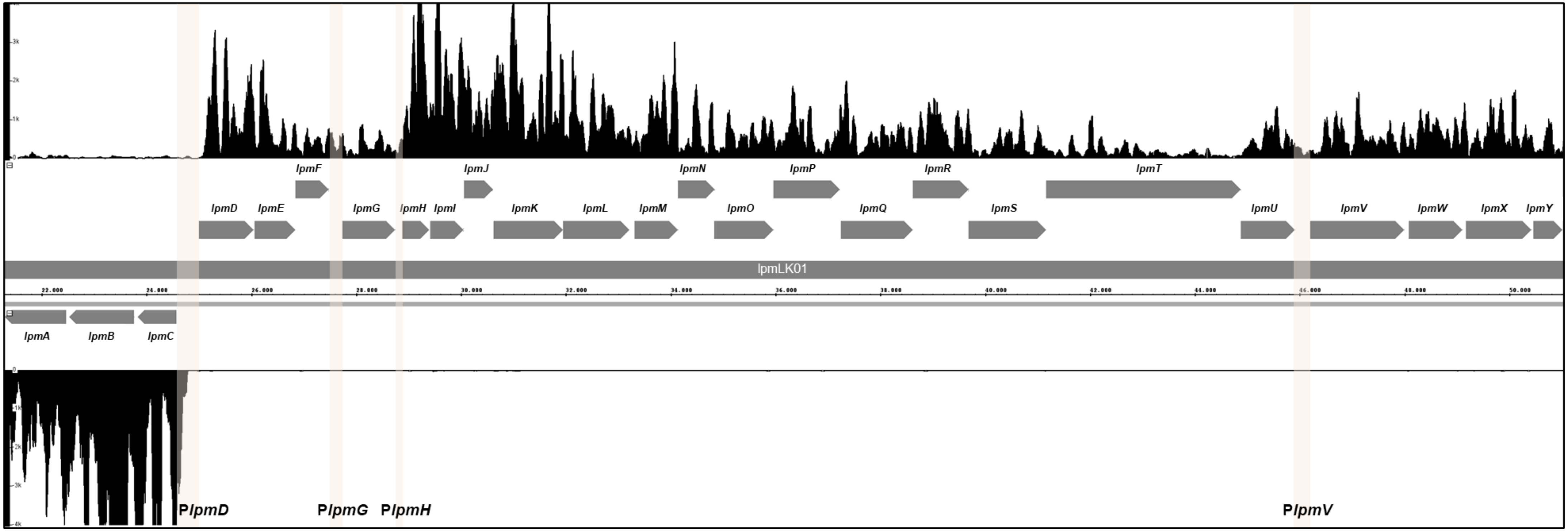

Figure S4: Transcription of the liposidomycin gene cluster in *S. coelicolor* M512/lpmLK01 after two days of cultivation. The genes of the cluster, depicted as arrows, are divided into sense and anti-sense strand and respective coverage plots are mapped against them. A threshold value of 4000 read counts is applied. Intergenic regions selected for DNA-affinity-capturing assays are labelled (P*lpmD*, P*lpmG*, P*lpmH* and P*lpmV*) and highlighted with light red bars.

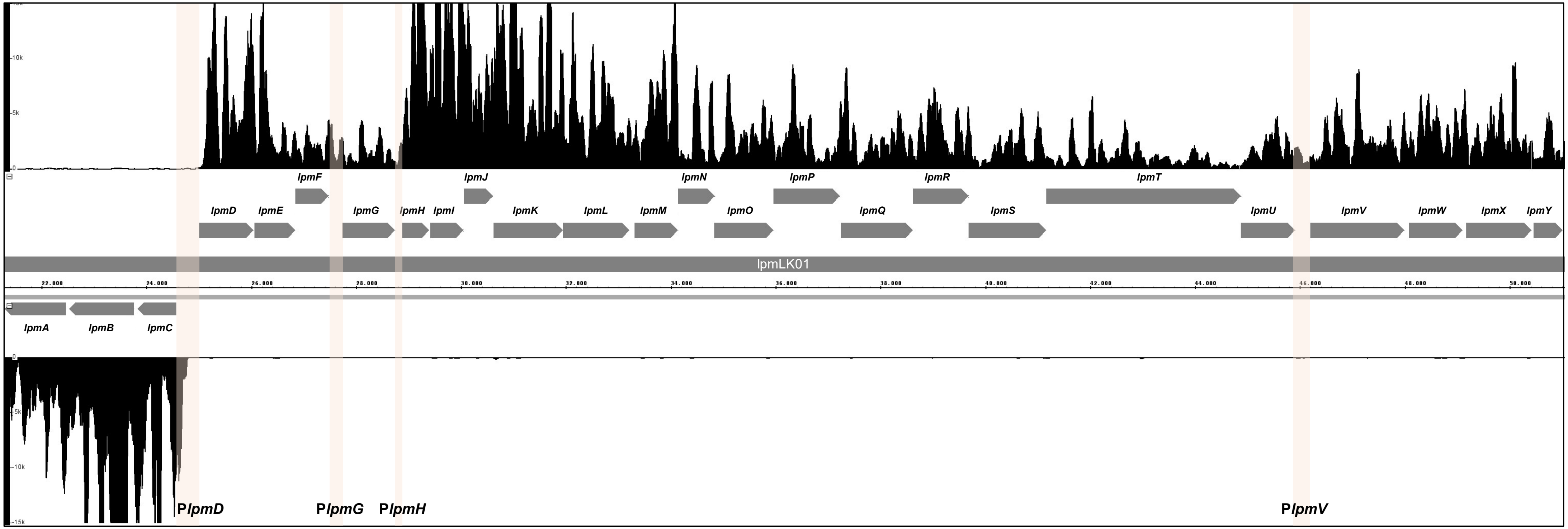

Figure S5: Transcription of the liposidomycin gene cluster in *S. coelicolor* M512/lpmLK01 after four days of cultivation. The genes of the cluster, depicted as arrows, are divided into sense and anti-sense strand and respective coverage plots are mapped against them. A threshold value of 15000 read counts is applied. Intergenic regions selected for DNA-affinity-capturing assays are labelled (P*lpmD*, P*lpmG*, P*lpmH* and P*lpmV*) and highlighted with light red bars.

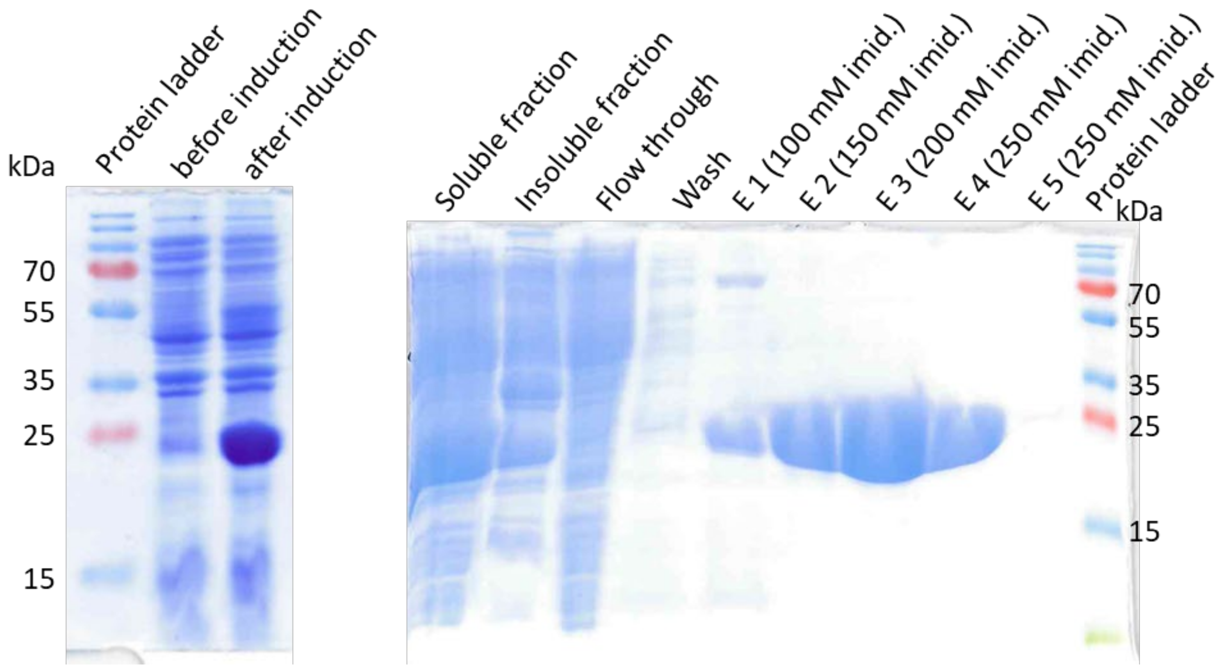

Figure S6: SDS gels of overexpression and purification of Sco4385 (calculated molecular weight: 24,69 kDa). Imid.: imidazole.

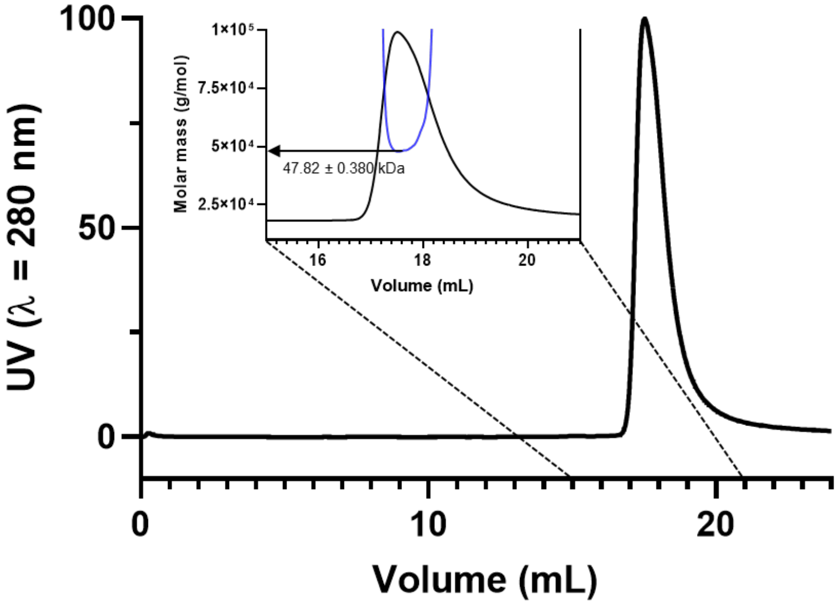

Figure S7: SEC-MALS elution profile of Sco4385. One single peak is detected in the elution profile after 43.823 min (equivalent to an elution volume of 17.53 mL) with an apparent molecular weight of 47.82 ± 0.38 kDa. This corresponds to a dimeric complex of Sco4385. Peak intensity was normalized to the single peak.

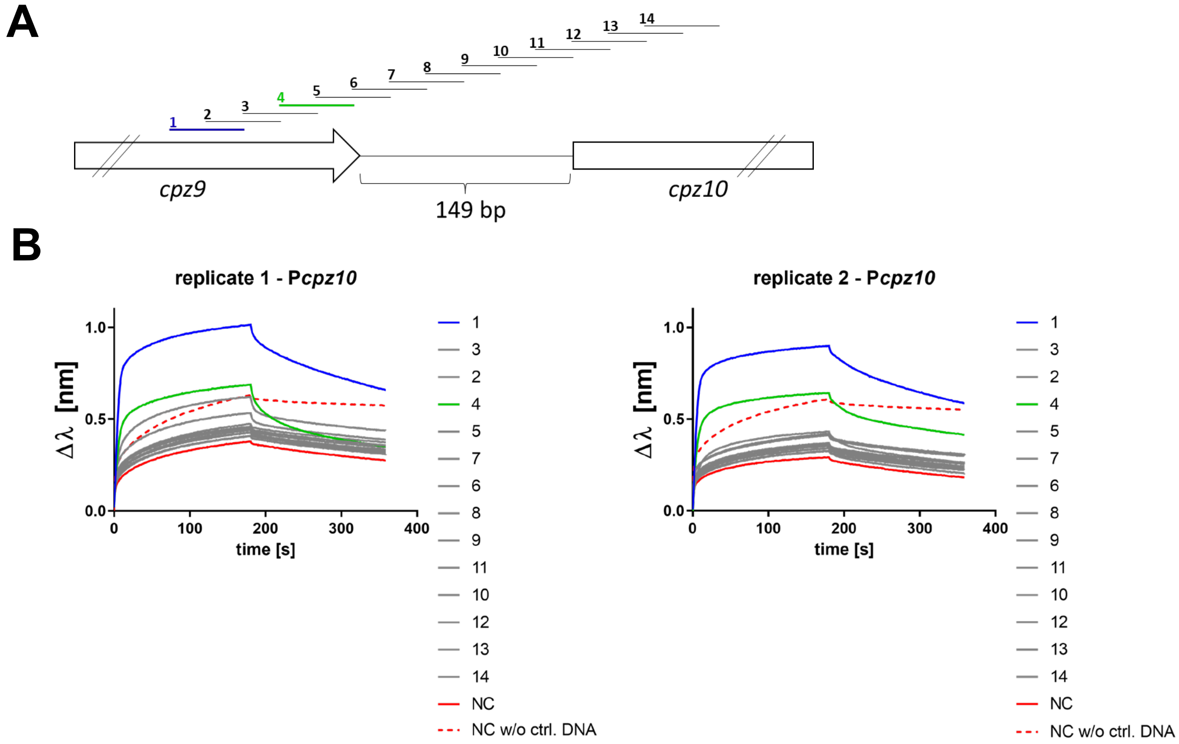

Figure S8: Distribution of the 50- bp segments on the extended P*cpz10* promoter region (A) and BLI binding curves of Sco4385 on all segments (B). NC: negative control. NC w/o ctrl. DNA: ReDCaT linker and Sco4385.

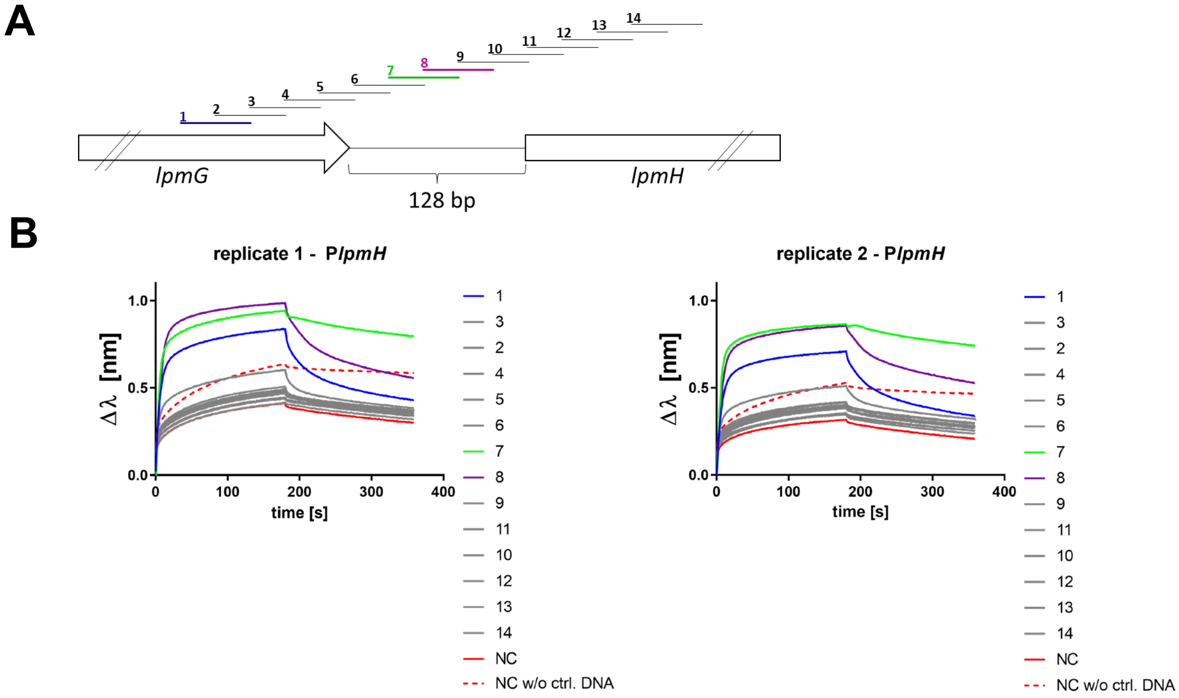

Figure S9: Distribution of the 50- bp segments on the extended P*lpmH* promoter region (A) and BLI binding curves of Sco4385 on all segments (B). NC: negative control. NC w/o ctrl. DNA: ReDCaT linker and Sco4385.

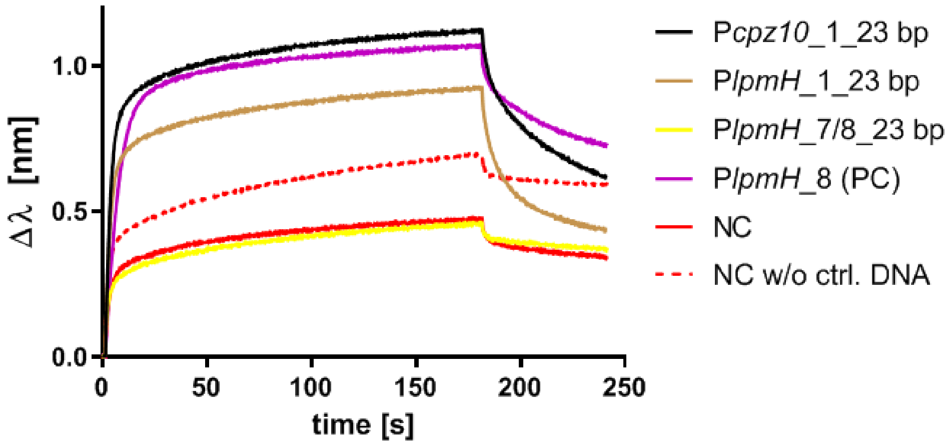

Figure S10: BLI binding curves of Sco4385 to the putative 23-bp binding sequences predicted by GLAM2. PC: positive control. NC: negative control. NC w/o ctrl. DNA: ReDCaT linker and Sco4385.

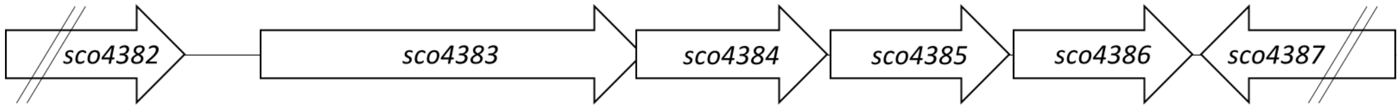

Gene	Putative function
sco4383	4-coumarate-CoA ligase
sco4384	Enoyl-CoA hydratase
sco4385	TetR-type regulator
sco4386	Hypothetical protein

Figure S11: Genetic organization of the putative gene cluster containing *sco4385* and predicted function of neighboring genes.

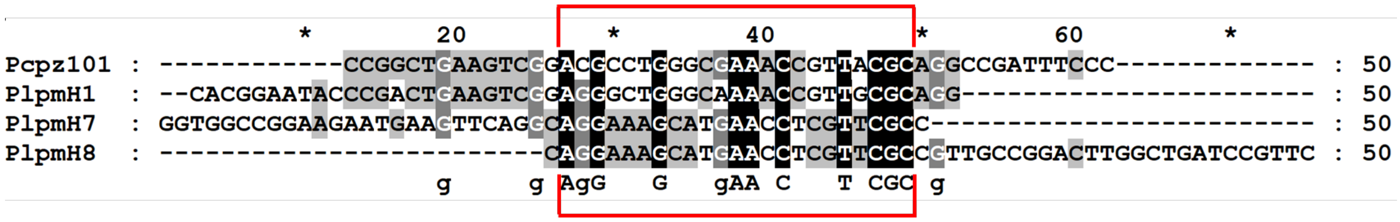

Figure S12: Sequence alignment of segments bound by Sco4385 (excluding P*cpz10* 4). The 23-bp region that was defined as the consensus sequence is highlighted in red brackets.

Table S1: Table of the wavelength-shift intensities of all measured samples (RAW data, P*cpz10* segments), numbering according to the segment number.

	1		2		3		4		5		6		7	
P*cpz10*	1.01	0.90	0.46	0.41	0.45	0.42	0.68	0.64	0.46	0.34	0.42	0.35	0.45	0.37
$\bar{x}$	0.95		0.43		0.43		0.66		0.40		0.39		0.41	
Std	0.06		0.02		0.02		0.02		0.06		0.04		0.04	

	8		9		10		11		12		13		14	
P*cpz10*	0.62	0.43	0.44	0.32	0.47	0.34	0.42	0.33	0.41	0.36	0.40	0.36	0.53	0.39
$\bar{x}$	0.52		0.38		0.40		0.37		0.38		0.38		0.46	
Std	0.09		0.06		0.07		0.05		0.02		0.02		0.07	

Table S2: Table of the wavelength-shift intensities of all measured samples (RAW data, P*lpmH* segments), numbering according to the segment number.

	1		2		3		4		5		6		7	
P*lpmH*	0.83	0.71	0.60	0.51	0.48	0.40	0.47	0.39	0.47	0.34	0.46	0.42	0.94	0.86
$\bar{x}$	0.77		0.55		0.44		0.43		0.40		0.44		0.90	
Std	0.06		0.05		0.04		0.04		0.06		0.02		0.04	

	8		9		10		11		12		13		14	
P*lpmH*	0.98	0.85	0.50	0.40	0.44	0.38	0.44	0.35	0.48	0.39	0.49	0.41	0.41	0.35
$\bar{x}$	0.92		0.45		0.41		0.39		0.43		0.45		0.38	
Std	0.06		0.05		0.03		0.04		0.05		0.04		0.03	

The mean all measurements of the negative control (P*hrdB* segment).

	NC			
	0.37	0.29	0.41	0.31
$\bar{x}$	0.35			
Std	0.05			

Table S3: Bacterial strains and plasmids used in this study.

**Strain**	**Relevant genetic characteristics**	**Reference**
E. coli BL21 (DE3)	strain B, *F⁻ ompT gal dcm lon* $hsdS_B(r_B^- m_B^-)$ *λ(DE3 [lacI lacUV5-T7p07 ind1 sam7 nin5])* $[malB^+]_{K-12}(\lambda^S)$	(1)
E. coli ET12567	Strain defective in DNA methylation, *dam⁻*, *dcm⁻*, *nsdM⁻*); Tet^R, Cml^R	(2)
E. coli XL1 blue	*recA1, endA1,gyrA96 (nalr), thi-1, hsdR17*$(rk^- mk^+)$*, supE44, relA1, lac [F´, proAB+, lacIqZ_M15, Tn10,(*Tet^R*)]*	Stratagene Inc., La Jolla, CA, USA
S. coelicolor M512	*SCP1−, SCP2−, ΔactII-ORF4, ΔredD*	(3)
S. coelicolor M512/cpzLK09	*S. coelicolor* M512 containing the caprazamycin gene cluster from *S. sp*. MK730-62F2, Kan^R	(4)
S. coelicolor M512/lpmLK01	*S. coelicolor* M512 containing the liposidomycin gene cluster from *S. sp*. SN1061-M, Kan^R	(5)
S. coelicolor M512/cpzLK09 Δ*sco3571*	*S. coelicolor* M512/cpzLK09 with marker- and scarless deletion of *sco3571*	This study
S. coelicolor M512/lpmLK01 Δ*sco3571*	*S. coelicolor* M512/lpmLK01 with marker- and scarless deletion of *sco3571*	This study
S. coelicolor M512/cpzLK09 Δ*sco4385*	*S. coelicolor* M512/cpzLK09 with marker- and scarless deletion of *sco4385*	This study
S. coelicolor M512/lpmLK01 Δ*sco4385*	*S. coelicolor* M512/lpmLK01 with marker- and scarless deletion of *sco4385*	This study
**Plasmid**	**Characteristics**	**Reference**
pBlueScript II SK (+)	$Carb^R$, general cloning vector	Stratagene Inc., La Jolla, CA, USA
pCRISPR-TT	$Apra^R$, carrying the CRISPR-Cas9 system; Cas9 under control of a theophylline-inducible riboswitch; temperature sensitive replication	Prof. Dr. Marta Mendes, IBMC, University of Porto, Portugal
pHis8	Kan^R, overexpression vector for proteins carrying a N-terminal 8x His-tag	(6)
pUWL-apra-oriT	$Carb^R$, $Apra^R$, constitutive *ermEp** promoter, pIJ101-origin, ColE1-origin, *oriT*	(7)
pUZ8002	Kan^R, carrying *tra* genes, RP4, for biparental conjugation	(8)
pSW4	$Apra^R$, pUWL-apra-oriT with *sco2987* (MarR) cloned into *Hind*III and *Spe*I sites	This study

pSW5	Apra^R<br>pUWL-apra-oriT with *sco3571* (CRP) cloned into *Hind*III and *Spe*I sites	This study
pSW6	Apra^R, pUWL-apra-oriT with *sco4385* (TetR) cloned into *Hind*III and *Spe*I sites	This study
pSW7	Apra^R, pUWL-apra-oriT with *sco5956* (TetR) cloned into *Hind*III and *Spe*I sites	This study
pSW8	Apra^R, pUWL-apra-oriT with *cpz9* cloned into *Hind*III and *Spe*I sites	This study
pSW9	Apra^R, pUWL-apra-oriT with *lpmG* cloned into *Hind*III and *Spe*I sites	This study
pSW18	Apra^R, pCRISPR-TT with spacer sequence for *sco4385* in *Nco*I and *Sna*BI sites and homology domains in *Stu*I site	This study
pSW20	Apra^R, pCRISPR-TT with spacer sequence for *sco3571* in *Nco*I and *Sna*BI sites and homology domains in *Stu*I site	This study
pSW21	Kan^R, pHis8 with *sco4385* (TetR) cloned into *Eco*RI and *Hind*III sites	This study

Table S4: Oligonucleotides used in this study.

Name	Sequence (5´ → 3´)	Application
lpmDp_fwd	CGATGTCGCTGTTCGCCTG	544 bp promoter fragment upstream of *lpmD* incl. 23 bp DAC biotin linker
lpmDp_rev	GAGGAGTCGTCGATGTGGAGACCC CTGTGTGGTGACCTTGTGACC	
lpmGp_fwd	GGATGGGAGCCGTTGAGCACG	520 bp promoter fragment upstream of *lpmG* incl. 23 bp DAC biotin linker
lpmGp_rev	GAGGAGTCGTCGATGTGGAGACCC TTCACCGATGCGCCGCACC	
lpmHp_fwd	CCTGCGCAACGGTTTTGCC	369 bp promoter fragment upstream of *lpmH* incl. 23 bp DAC biotin linker
lpmHp_rev	GAGGAGTCGTCGATGTGGAGACCG TGCTGGAGGGCGAGTTGCC	
lpmVp_fwd	CCGACGGCATCCATCTGACG	455 bp promoter fragment upstream of *lpmV* incl. 23 bp DAC biotin linker
lpmVp_rev	GAGGAGTCGTCGATGTGGAGACCG ACTCGTACCACCGCAACACG	
cpz6p_fwd	GAGGAGTCGTCGATGTGGAGACCC AGGAAGAACTCGGTTGTGC	424 bp promoter fragment upstream of *cpz6* incl. 23 bp DAC biotin linker
cpz6p_rev	GCTGTTGTGACCTTGTTGC	

Name	Sequence (5´ → 3´)	Application
cpz9p_fwd	GAGGAGTCGTCGATGTGGAGACCG CTGGGAGCCATTGTGCGC	358 bp promoter fragment upstream of *cpz9* incl. 23 bp DAC biotin linker
cpz9p_rev	CGGCCACCCTCCTGTCTTTGC	
cpz10p_fwd	GAGGAGTCGTCGATGTGGAGACCG GGAAATCGGCCTGCGTAACG	365 bp promoter fragment upstream of *cpz10* incl. 23 bp DAC biotin linker
cpz10p_rev	CGGATTCGCGGAGCTTGTCG	
cpz24p_fwd	GAGGAGTCGTCGATGTGGAGACCC CGACGGCATCCATCTGACG	431 bp promoter fragment upstream of *cpz24* incl. 23 bp DAC biotin linker
cpz24p_rev	CACGTCCCGCTGTCTGTCC	
hrdBp_fwd	GTCAACTTCTGACCGTCCAC	559 bp promoter fragment upstream of *hrdB* incl. 23 bp DAC biotin linker
hrdBp_rev	GAGGAGTCGTCGATGTGGAGACCA ATGAGCGCCATGACAGAG	
DAC Biotin	Biotin-GAGGAGTCGTCGATGT GGAGACC	Linker sequence for attachment of the biotin-tag to amplified promoter fragments
qPCRcpz10_fwd	CTTCCGTTCAAGCCCATTC	qPCR primers for detection of *cpz10* transcripts (156 bp)
qPCRcpz10_rev	AATGTGCAGCACCTTGTC	
qPCRlpmH_fwd	AGCCTGTTGGAAAAGCATC	qPCR primers for detection of *lpmH* transcripts (121 bp)
qPCRlpmH_rev	CCGAGGAAATCAACGACTG	
qPCRhrdB_fwd	TGACGCTGATGGTCAGTGC	qPCR primers for detection of *hrdB* transcripts (124 bp)
qPCRhrdB_rev	GTCGCCTTCCTGCTGGTC	
sco4385_HindIII_fwd	aaaAAGCTTGTGAACGCGGCCGACC GCACG	Amplification of *sco4385* and attachment of HindIII and SpeI restriction site for cloning (621 bp)
sco4385_SpeI_rev	aaaACTAGTTCACTGTGCCAGCGCG GCGTCC	
sco5956_HindIII_fwd	aaaAAGCTTGTGACGGCACCTGCCA CGGCC	Amplification of *sco5956* and attachment of HindIII and SpeI restriction site for cloning (825 bp)
sco5956_SpeI_rev	aaaACTAGTCTACTCGGCCGGGACG GACTTCG	

**Name**	**Sequence (5´ → 3´)**	**Application**
sco2987_HindIII_fwd	aaaAAGCTTATGACCACGCCCGCTACCGAG	Amplification of *sco2987* and attachment of HindIII and SpeI restriction site for cloning (519 bp)
sco2987_SpeI_rev	aaaACTAGTTCAGGACGGTTCGGGTGCCG	
sco3571_HindIII_fwd	aaaAAGCTTGTGGACGACGTTCTGCGGCG	Amplification of *sco3571* and attachment of HindIII and SpeI restriction site for cloning (687 bp)
sco3571_SpeI_rev	aaaACTAGTTCAGCGGGAGCGCTTGGCC	
cpz9_HindIII_fwd	aaaAAGCTTGTGATCTTCCAGGCGTCACCGAC	Amplification of *cpz9* and attachment of *Hind*III and *Spe*I restriction site for cloning (1059 bp)
cpz9_SpeI_rev	aaaACTAGTCTATTGACTGATCGCGCCCCACC	
lpmG_HindIII_fwd	aaaAAGCTTGTGTGGAAACGGGGGTTTGG	Amplification of *lpmG* and attachment of *Hind*III and *Spe*I restriction site for cloning (1023 bp)
lpmG_SpeI_rev	aaaACTAGTTTAGCCGACCGCGTCCCG	
pUWL_test_fwd	ACGCCTGGTCGATGTCGGAC	Test- and sequencing primers for cloning into the MCS of pUWL-apra-oriT vector
pUWL_new_rev	GAGCGAGGAAGCGGAAGAGC	
pCRISPR-TT_sco3571_fwd	CATGCCATGG**TCGGGGCGGACGTTCAGCCA** GTTTTAGAGCTAGAAATAGC*	Amplification of the sgRNA sequence with insertion of the spacer sequence for *sco3571* deletion (123 bp)
pCRISPR-TT_sco4385_fwd	CATGCCATGG**CAGGCCCTGGACGGTTTCCC** GTTTTAGAGCTAGAAATAGC*	Amplification of the sgRNA sequence with insertion of the spacer sequence for *sco4385* deletion (123 bp)
pCRISPR-TT_rev	ACGCCTACGTAAAAAAAGCACCGACTCGGTGCC	Universal reverse primer for amplification of the sgRNAs
sco3571_HD_upstream_fwd	CCCCCGGGCTGCAGGAATTCGATATCGTCGGGTACTTGGCGAAGAGG	Amplification of upstream homology domain of *sco3571* (942 bp) and assembly in pBlueScript SK II
sco3571_HD_upstream_rev	CCCGGGGAGACCCCAGGGGGGAGTTCTCTCCTTGTCGACCGG	
sco3571_HD_downstream_fwd	GGTCGACAAGGAGAGAACTCCCCCCTGGGGTCTCCCCGG	Amplification of downstream homology domain of *sco3571* (935

Name	Sequence (5´ → 3´)	Application
sco3571_HD_ downstream_rev	TCGACGGTATCGATAAGCTTGATATCCGGCGTCCTGTCGGGACCG	bp) and assembly in pBlueScript SK II
sco4385_HD_ upstream_fwd	CCCCCGGGCTGCAGGAATTCGATATCCTTCCAGGTGGCCCCCGCCGAA	Amplification of upstream homology domain of *sco4385* (1074 bp) and assembly in pBlueScript SK II
sco4385_HD_ upstream_rev	GCCCCGGTGCCGGACGCCGGGGCGCCGCTCACACCACCCATT	
sco4385_HD_down_ fwd	CCGGCGTCCGGCACCGGG	Amplification of downstream homology domain of *sco4385* (1079 bp) and assembly in pBlueScript SK II
sco4385_HD_down_ rev	TCGACGGTATCGATAAGCTTGATATCGCGCGCGAGCTGGACGCG	
pSET152_test_fwd	ACGCCAGGGTTTTCCCAGTCAC	Test- and sequencing primers for cloning into the MCS of pBlueScript SK II vector
pSET152_test_rev	AGCTGGCACGACAGGTTTCCC	
pCRISPR-TT_test_Stu_fwd	GATCCACCAGAGCATCACCG	Test- and sequencing primers for cloning the homology domains into StuI restriction site of pCRISPR-TT-spacer
pCRISPR-TT_test_Stu_rev	GTCGACGCGCTGTTCTCTCG	
sco4385_KO_test_fwd	ATCGACGAGGAGGGCTGGCTG	Test- and sequencing primers for validation of the deletion of *sco4385*
sco4385_KO_test_rev	CATGGTGGTCTCCACGGCGGA	
sco3571_KO_test_ fwd	CCGGAAGAAGCCGGTCGGAC	Test- and sequencing primers for validation of the deletion of *sco3571*
sco3571_KO_test_ rev	CAAGCACGTCCCGAGCACGG	
sco4385_EcoRI_fwd	aaaGAATTCGTGAACGCGGCCGACCGCACG	Amplification of *sco4385* (621 bp) and attachment of EcoRI and HindIII restriction site for cloning into the pHis8 vector
sco4385_HindIII_rev	aaaAAGCTTTCACTGTGCCAGCGCGGCGTCC	

Restriction sites for enzymes are underlined.

* Spacer sequences to be inserted into pCRISPR-TT are bold.

Table S5: Oligonucleotides used for BLI measurements. Forward and reverse oligonucleotides were annealed prior to BLI measurements and bound to the ReDCaT linker via the single stranded overhang attached to the reverse oligonucleotide sequence.

Name	Sequence (5´ → 3´)
ReDCaT linker	Biotin-GCAGGAGGACGTAGGGTAGG
PhrdB_NC_fwd	CTGTGCATCTCCCCGGCCCGCCCGCACCGTCGGCCCATTCCCAAGCCGGT
PhrdB_NC_rev	ACCGGCTTGGGAATGGGCCGACGGTGCGGGCGGGCCGGGGAGATGCACAGcctacc ctacgtcctcctgc
Pcpz10_1_fwd	GGGAAATCGGCCTGCGTAACGGTTTCGCCCAGGCGTCCGACTTCAGCCGG
Pcpz10_1_rev	CCGGCTGAAGTCGGACGCCTGGGCGAAACCGTTACGCAGGCCGATTTCCCcctaccta cgtcctcctgc
Pcpz10_2_fwd	CGCCCAGGCGTCCGACTTCAGCCGGGTGTTTCGTGGCCGCTACGGTGTCC
Pcpz10_2_rev	GGACACCGTAGCGGCCACGAAACACCCGGCTGAAGTCGGACGCCTGGGCGcctaccct acgtcctcctgc
Pcpz10_3_fwd	GTGTTTCGTGGCCGCTACGGTGTCCCGCCGGGTAAATTCCGCGACGACTG
Pcpz10_3_rev	CAGTCGTCGCGGAATTTACCCGGCGGGACACCGTAGCGGCCACGAAACACcctaccta cgtcctcctgc
Pcpz10_4_fwd	CGCCGGGTAAATTCCGCGACGACTGGTTCCGGTGGGGCGCGATCAGTCAA
Pcpz10_4_rev	TTGACTGATCGCGCCCCACCGGAACCAGTCGTCGCGGAATTTACCCGGCGcctaccta cgtcctcctgc
Pcpz10_5_fwd	GTTCCGGTGGGGCGCGATCAGTCAATAGCCGGTCGCATGAGGACAATGAC
Pcpz10_5_rev	GTCATTGTCCTCATGCGACCGGCTATTGACTGATCGCGCCCCACCGGAACcctaccctacg tcctcctgc
Pcpz10_6_fwd	TAGCCGGTCGCATGAGGACAATGACAGAGCCCGAAGTGACCGGGAAAATA
Pcpz10_6_rev	TATTTTCCCGGTCACTTCGGGCTCTGTCATTGTCCTCATGCGACCGGCTAcctaccctacgt cctcctgc
Pcpz10_7_fwd	AGAGCCCGAAGTGACCGGGAAAATAGTGTGAGACAGGGAGCGTTCGACCT
Pcpz10_7_rev	AGGTCGAACGCTCCCTGTCTCACACTATTTTCCCGGTCACTTCGGGCTCTcctaccctacg tcctcctgc
Pcpz10_8_fwd	GTGTGAGACAGGGAGCGTTCGACCTCGCTCGCCCTTGAGTCCCCAGGTGG
Pcpz10_8_rev	CCACCTGGGGACTCAAGGGCGAGCGAGGTCGAACGCTCCCTGTCTCACACcctaccct acgtcctcctgc
Pcpz10_9_fwd	CGCTCGCCCTTGAGTCCCCAGGTGGCTCGTCCATTCCTGCCAGGACATCA
Pcpz10_9_rev	TGATGTCCTGGCAGGAATGGACGAGCCACCTGGGGACTCAAGGGCGAGCGcctaccct acgtcctcctgc

Name	Sequence (5´ → 3´)
Pcpz10_10_fwd	CTCGTCCATTCCTGCCAGGACATCAACGGCGGAGCAGAGAAGGCGCGCCG
Pcpz10_10_rev	CGGCGCGCCTTCTCTGCTCCGCCGTTGATGTCCTGGCAGGAATGGACGAGcctaccctacgtcctcctgc
Pcpz10_11_fwd	ACGGCGGAGCAGAGAAGGCGCGCCGCTGTGCCCGGCCGTCGAAAGGCTTG
Pcpz10_11_rev	CAAGCCTTTCGACGGCCGGGCACAGCGGCGCGCCTTCTCTGCTCCGCCGTcctaccctacgtcctcctgc
Pcpz10_12_fwd	CTGTGCCCGGCCGTCGAAAGGCTTGCTTCGTGACAGCACTCACGTCCAGG
Pcpz10_12_rev	CCTGGACGTGAGTGCTGTCACGAAGCAAGCCTTTCGACGGCCGGGCACAGcctaccctacgtcctcctgc
Pcpz10_13_fwd	CTTCGTGACAGCACTCACGTCCAGGACCGAACTCGACATCGACCCCGACA
Pcpz10_13_rev	TGTCGGGGTCGATGTCGAGTTCGGTCCTGGACGTGAGTGCTGTCACGAAGcctaccctacgtcctcctgc
Pcpz10_14_fwd	ACCGAACTCGACATCGACCCCGACAAGCTCCGCGAATCCGTCGTGGAGTT
Pcpz10_14_rev	AACTCCACGACGGATTCGCGGAGCTTGTCGGGGTCGATGTCGAGTTCGGTcctaccctacgtcctcctgc
PlpmH_1_fwd	CCTGCGCAACGGTTTTGCCCAGCCCTCCGACTTCAGTCGGGTATTCCGTG
PlpmH_1_rev	CACGGAATACCCGACTGAAGTCGGAGGGCTGGGCAAAACCGTTGCGCAGGcctaccctacgtcctcctgc
PlpmH_2_fwd	TCCGACTTCAGTCGGGTATTCCGTGCCAATTACGGCATACCGCCGGGCAA
PlpmH_2_rev	TTGCCCGGCGGTATGCCGTAATTGGCACGGAATACCCGACTGAAGTCGGAcctaccctacgtcctcctgc
PlpmH_3_fwd	CCAATTACGGCATACCGCCGGGCAAGTTTCGGGACGACTGGTTCCGGCGG
PlpmH_3_rev	CCGCCGGAACCAGTCGTCCCGAAACTTGCCCGGCGGTATGCCGTAATTGGcctaccctacgtcctcctgc
PlpmH_4_fwd	GTTTCGGGACGACTGGTTCCGGCGGGACGCGGTCGGCTAACCAGGTGGTC
PlpmH_4_rev	GACCACCTGGTTAGCCGACCGCGTCCCGCCGGAACCAGTCGTCCCGAAACcctaccctacgtcctcctgc
PlpmH_5_fwd	GACGCGGTCGGCTAACCAGGTGGTCGCTTCACGGCAATGACACATCCTGC
PlpmH_5_rev	GCAGGATGTGTCATTGCCGTGAAGCGACCACCTGGTTAGCCGACCGCGTCcctaccctacgtcctcctgc
PlpmH_6_fwd	GCTTCACGGCAATGACACATCCTGCGGTGGCCGGAAGAATGAAGTTCAGG
PlpmH_6_rev	CCTGAACTTCATTCTTCCGGCCACCGCAGGATGTGTCATTGCCGTGAAGCcctaccctacgtcctcctgc

Name	Sequence (5´ → 3´)
PlpmH_7_fwd	GGTGGCCGGAAGAATGAAGTTCAGGCAGGAAAGCATGAACCTCGTTCGCC
PlpmH_7_rev	GGCGAACGAGGTTCATGCTTTCCTGCCTGAACTTCATTCTTCCGGCCACCcctaccctacgtcctcctgc
PlpmH_8_fwd	CAGGAAAGCATGAACCTCGTTCGCCGTTGCCGGACTTGGCTGATCCGTTC
PlpmH_8_rev	GAACGGATCAGCCAAGTCCGGCAACGGCGAACGAGGTTCATGCTTTCCTGcctaccctacgtcctcctgc
PlpmH_9_fwd	GTTGCCGGACTTGGCTGATCCGTTCCGCAGGGGGAATTACTCCGTGACAG
PlpmH_9_rev	CTGTCACGGAGTAATTCCCCCTGCGGAACGGATCAGCCAAGTCCGGCAACcctaccctacgtcctcctgc
PlpmH_10_fwd	CGCAGGGGGAATTACTCCGTGACAGTACTGACGTCCAGGACCGTGCTCGA
PlpmH_10_rev	TCGAGCACGGTCCTGGACGTCAGTACTGTCACGGAGTAATTCCCCCTGCGcctaccctacgtcctcctgc
PlpmH_11_fwd	TACTGACGTCCAGGACCGTGCTCGACATCGACCCGGTCAGGCTCCGCGAA
PlpmH_11_rev	TTCGCGGAGCCTGACCGGGTCGATGTCGAGCACGGTCCTGGACGTCAGTAcctaccctacgtcctcctgc
PlpmH_12_fwd	CATCGACCCGGTCAGGCTCCGCGAATCCGTGGCAAGCCTGTTGGAAAAGC
PlpmH_12_rev	GCTTTTCCAACAGGCTTGCCACGGATTCGCGGAGCCTGACCGGGTCGATGcctaccctacgtcctcctgc
PlpmH_13_fwd	TCCGTGGCAAGCCTGTTGGAAAAGCATCCGTTGGTATTCGAGGGCACACG
PlpmH_13_rev	CGTGTGCCCTCGAATACCAACGGATGCTTTTCCAACAGGCTTGCCACGGAcctaccctacgtcctcctgc
PlpmH_14_fwd	ATCCGTTGGTATTCGAGGGCACACGGCAACTCGCCCTCCAGCACCGGTCG
PlpmH_14_rev	CGACCGGTGCTGGAGGGCGAGTTGCCGTGTGCCCTCGAATACCAACGGATcctaccctacgtcctcctgc
Pcpz10_1_Konsensus_fwd	ACGCCTGGGCGAAACCGTTACGC
Pcpz10_1_Konsensus_rev	GCGTAACGGTTTCGCCCAGGCGTcctaccctacgtcctcctgc
PlpmH_1_Konsensus_fwd	AGGGCTGGGCAAAACCGTTGCGC
PlpmH_1_Konsensus_rev	GCGCAACGGTTTTGCCCAGCCCTcctaccctacgtcctcctgc
PlpmH_7/8_Konsensus_fwd	AGGAAAGCATGAACCTCGTTCGC

**Name**	**Sequence (5´ → 3´)**
PlpmH_7/8_Konsensus_rev	GCGAACGAGGTTCATGCTTTCCTcctaccctacgtcctcctgc

Table S6: LFQ values of bound proteins detected by DNA-affinity capturing assay (DACA). Listed are proteins binding to the corresponding promoters of both clusters, the CRP regulator Sco3571, the CSR of the Cpz BGC, Cpz9, and examples of global regulators of secondary metabolism in *Streptomyces*.

**Protein name/Sco No.**	**Regulator family**	**P*cpz6***		**P*cpz9***		**P*cpz10***		**P*cpz24***		**P*hrdB* (Cpz DACA)**		**P*lpmD***		**P*lpmG***		**P*lpmH***		**P*lpmV***		**P*hrdB* (Lpm DACA)**	
		**36 h**	**54 h**	**36 h**	**54 h**	**36 h**	**54 h**	**36 h**	**54 h**	**36 h**	**54 h**	**48 h**	**72 h**	**48 h**	**72 h**	**48 h**	**72 h**	**48 h**	**72 h**	**48 h**	**72 h**
Sco2987	MarR		1.35 E+08									3.41 E+08	7.52 E+08								
Sco4385	TetR					4.87 E+08	1.48 E+08									5.77 E+09	4.66 E+09				
Sco5956	TetR					1.02 E+08										2.39 E+08					
Sco3571	CRP	3.86 E+09	7.61 E+09	4.74 E+09	7.46 E+09	4.55 E+09	7.07 E+09	5.54 E+09	9.23 E+09	4.42 E+09	8.22 E+09	4.05 E+09	1.52 E+09	4.45 E+09	1.36 E+09	3.86 E+09	1.45 E+09	4.49 E+09	1.30 E+09	6.35 E+09	2.58 E+09
Cpz9	AraC	9.68 E+08	2.73 E+09	3.88 E+08	4.00 E+08	5.39 E+08	1.24 E+09	7.00 E+08	6.01 E+08		3.07 E+08	Not determined									
AdpA (Sco2792)	AraC	2.50 E+11	2.76 E+10	1.09 E+11	1.18 E+10	1.39 E+11	2.16 E+10	1.03 E+11	8.49 E+09	3.42 E+11	3.53 E+10	2.08 E+11	8.39 E+10	1.31 E+11	6.47 E+10	1.00 E+11	8.58 E+10	2.25 E+10	1.46 E+10	1.83 E+11	4.89 E+10
NdgR (Sco5552)	IclR	1.45 E+11	4.35 E+10	7.71 E+10	1.69 E+10	1.15 E+11	4.07 E+10	7.68 E+10	1.26 E+10	1.25 E+11	2.33 E+10	3.68 E+10	1.40 E+10	4.21 E+10	1.58 E+10	3.75 E+10	1.44 E+10	4.75 E+10	2.90 E+10	3.46 E+09	1.11 E+09
SlbR (Sco0608)	γ-butyrolactone-binding regulator	1.55 E+10		1.75 E+10		1.56 E+10		7.61 E+08		3.09 E+08		2.40 E+10		1.62 E+10		1.60 E+10		2.16 E+10		1.09 E+09	
Rok7B7 (Sco6008)	ROK	1.71 E+09	6.42 E+08	5.89 E+08	1.08 E+08	2.31 E+08	2.20 E+08	2.00 E+08	9.80 E+07			7.87 E+08	1.06 E+09	5.70 E+08	5.36 E+08	6.85 E+08	7.23 E+08			1.86 E+08	
AtrA (Sco4118)	TetR			3.11 E+08	2.68 E+08					5.78 E+09	3.66 E+09	2.64 E+09	1.77 E+09	9.78 E+08	9.97 E+08	3.91 E+09	2.42 E+09	7.61 E+07		2.54 E+09	1.55 E+09

Table S7: Values of wavelength-shifts resulting from Sco4385 binding to selected promoter segments and the putative consensus sequences predicted by GLAM2. NC: negative control.

Promoter	Segment	Δλ by Sco4385 binding
P*cpz10*	1	0.95
	P*cpz10*_1_23 bp	1.12
P*lpmH*	1	0.77
	P*lpmH*_1_23 bp	0.93
	7	0.90
	8	1.07
	P*lpmH*_7/8_23 bp	0.46
P*hrdB*	NC	0.48

References

1. Studier WF, Rosenberg AH, Dunn JJ, Dubendorff JW. 1990. Use of T7 RNA polymerase to direct expression of cloned genes, p. 60–89. *In* Methods in Enzymology.

2. MacNeil DJ, Gewain KM, Ruby CL, Dezeny G, Gibbons PH, Maeneil T. 1992. Analysis of *Streptomyces avermitilis* genes required for avermectin biosynthesis utilizing a novel integration vector. Gene 111:61–68.

3. Floriano B, Bibb M. 1996. *afsR* is a pleiotropic but conditionally required regulatory gene for antibiotic production in *Streptomyces coelicolor* A3(2). Mol Microbiol 21:385–396.

4. Kaysser L, Lutsch L, Siebenberg S, Wemakor E, Kammerer B, Gust B. 2009. Identification and manipulation of the caprazamycin gene cluster lead to new simplified liponucleoside antibiotics and give insights into the biosynthetic pathway. Journal of Biological Chemistry 284:14987–14996.

5. Kaysser L, Siebenberg S, Kammerer B, Gust B. 2010. Analysis of the liposidomycin gene cluster leads to the identification of new caprazamycin derivatives. ChemBioChem 11:191–196.

6. Jez JM, Ferrer J-L, Bowman ME, Dixon RA, Noel JP. 2000. Dissection of malonyl-coenzyme A decarboxylation from polyketide formation in the reaction mechanism of a plant polyketide synthase. Biochemistry 39:890–902.

7. Erb A, Luzhetskyy A, Hardter U, Bechthold A. 2009. Cloning and sequencing of the biosynthetic gene cluster for saquayamycin Z and galtamycin B and the elucidation of the assembly of their saccharide chains. ChemBioChem 10:1392–1401.

8. Paget MSB, Chamberlin L, Atrih A, Foster SJ, Buttner MJ. 1999. Evidence that the extracytoplasmic function Sigma Factor sigmaE is required for normal cell wall structure in *Streptomyces coelicolor* A3(2). J Bacteriol 181:204–211.
